# Lignocellulolytic Potential of Microbial Consortia Isolated from a Local Biogas Plant: The Case of Thermostable Xylanases Secreted by Mesophilic Bacteria

**DOI:** 10.3390/ijms25021090

**Published:** 2024-01-16

**Authors:** Luca Bombardi, Andrea Salini, Martina Aulitto, Luca Zuliani, Marco Andreolli, Paola Bordoli, Annalaura Coltro, Nicola Vitulo, Claudio Zaccone, Silvia Lampis, Salvatore Fusco

**Affiliations:** 1 Biochemistry and Industrial Biotechnology (BIB) Laboratory, Department of Biotechnology, University of Verona, 37134 Verona, Italy; luca.bombardi@univr.it (L.B.); andrea.salini@univr.it (A.S.); zuliani.luca@outlook.com (L.Z.); paolabordoli98@gmail.com (P.B.); annalaura.coltro@studenti.univr.it (A.C.); 2 Department of Biology, University of Naples Federico II, 80126 Naples, Italy; martina.aulitto@unina.it; 3 Lab of Environmental Microbiology & VUCC-DBT Verona University Culture Collection, Laboratory, Department of Biotechnology, University of Verona, 37134 Verona, Italy; marco.andreolli@univr.it (M.A.); silvia.lampis@univr.it (S.L.); 4 Computational Genomics Laboratory, Department of Biotechnology, University of Verona, 37134 Verona, Italy; nicola.vitulo@univr.it; 5 Lab of Soil and Biomass Chemistry, Department of Biotechnology, University of Verona, 37134 Verona, Italy; claudio.zaccone@univr.it

**Keywords:** carbohydrate-active enzymes, spent mushroom substrate, microbial diversity, microbiome dynamics, microbiomes, metagenomic analyses, biomass conversion, biorefineries, total carbohydrate analysis, thermal analysis

## Abstract

Lignocellulose biomasses (LCB), including spent mushroom substrate (SMS), pose environmental challenges if not properly managed. At the same time, these renewable resources hold immense potential for biofuel and chemicals production. With the mushroom market growth expected to amplify SMS quantities, repurposing or disposal strategies are critical. This study explores the use of SMS for cultivating microbial communities to produce carbohydrate-active enzymes (CAZymes). Addressing a research gap in using anaerobic digesters for enriching microbiomes feeding on SMS, this study investigates microbial diversity and secreted CAZymes under varied temperatures (37 °C, 50 °C, and 70 °C) and substrates (SMS as well as pure carboxymethylcellulose, and xylan). Enriched microbiomes demonstrated temperature-dependent preferences for cellulose, hemicellulose, and lignin degradation, supported by thermal and elemental analyses. Enzyme assays confirmed lignocellulolytic enzyme secretion correlating with substrate degradation trends. Notably, thermogravimetric analysis (TGA), coupled with differential scanning calorimetry (TGA-DSC), emerged as a rapid approach for saccharification potential determination of LCB. Microbiomes isolated at mesophilic temperature secreted thermophilic hemicellulases exhibiting robust stability and superior enzymatic activity compared to commercial enzymes, aligning with biorefinery conditions. PCR-DGGE and metagenomic analyses showcased dynamic shifts in microbiome composition and functional potential based on environmental conditions, impacting CAZyme abundance and diversity. The meta-functional analysis emphasised the role of CAZymes in biomass transformation, indicating microbial strategies for lignocellulose degradation. Temperature and substrate specificity influenced the degradative potential, highlighting the complexity of environmental–microbial interactions. This study demonstrates a temperature-driven microbial selection for lignocellulose degradation, unveiling thermophilic xylanases with industrial promise. Insights gained contribute to optimizing enzyme production and formulating efficient biomass conversion strategies. Understanding microbial consortia responses to temperature and substrate variations elucidates bioconversion dynamics, emphasizing tailored strategies for harnessing their biotechnological potential.

## 1. Introduction

Lignocellulose biomasses (LCB) are abundant and renewable resources, and as such, they have received growing attention as a potential feedstock to produce biofuels, chemicals, and other value-added products [1,2,3]. Among these, spent mushroom substrate (SMS) is a lignocellulose-rich agricultural leftover, resulting from mushrooms cultivation. SMS typically consists of a mixture of straw, sawdust, and other organic materials [4]. Given that mushrooms are popularly included in daily meals worldwide, due to their unique flavours and excellent nutritional content, their global cultivation and consumption have been steadily increasing, with a predicted market volume of 20.84 million tons by 2026 [5]. It is estimated that for every kilogram of fresh mushrooms produced, approximately 5 kg of SMS is generated [5]. Therefore, with this rapid expansion of the mushrooms industry, there is a large amount of SMS annually generated after mushrooms harvesting. Improper disposal of this by-product leads to environmental pollution, including air and water pollution, as well as soil contamination [6]. Efforts have been made to develop strategies for its efficient utilization; these include the use of SMS as an alternative to fertilisers and soil amendments (e.g., as vermicompost) and animal feed. Another viable option is to recycle the spent substrate by supplementing it with additional nutrients for a new cultivation cycle of some mushroom species. SMS can also serve as a feedstock to produce various types of biofuels, such as bioethanol [7], biogas, biohydrogen, and bio-oil, as well as volatile fatty acids (VFAs) [4,8].

Given its high residual content of cellulose and hemicellulose (approximately 57%, dry weight bases), another biotechnological application of SMS is its use as a feedstock to cultivate fungi in solid-state fermentation (SSF) to produce carbohydrate-active enzymes (CAZymes) [9,10,11,12]. These participate in the synthesis and breakdown of complex carbohydrates and glycoconjugates, serving various biological processes. They are classified into different families, including glycoside hydrolase (GH), glycosyl transferase (GT), carbohydrate-binding module (CBM), carbohydrate esterase (CE), auxiliary activities (AA), and polysaccharide lyase (PL). Among them, lignocellulolytic GHs, such as cellulases and hemicellulases, play a crucial role in the depolymerization of lignocellulose; CBMs are pivotal for the binding of cellulolytic enzymes to their specific substrates; AAs are associated with the degradation of lignin polymers, while CEs are essential for optimal hemicellulolytic activity. The production of CAZymes from SMS has been mainly performed with the micromycetes *Trichoderma* spp. or *Aspergillus* spp. [9,10] that are well-known producers of industrially relevant enzymes such as amylases, cellulases, and hemicellulases. For instance, such enzymes are essential for biofuel manufacturing, with amylases mainly used for the manufacturing of first-generation (1G) bioethanol, whereas cellulases and xylanases are employed for the saccharification of LCB to produce second-generation (2G) bioethanol, as well as other bioproducts [7].

Several studies have shown that numerous organisms, including bacteria, fungi, yeast, algae, snails, and crustaceans, produce xylanases [13]. However, industrial xylanase production is reported to be carried out mostly in fungi and bacteria. Xylanases have distinctive characteristics depending on the biological sources from which they have been isolated; therefore, they can be potentially used for several industrial applications. Some bacterial strains (including *Bacillus* sp.) have been reported to produce xylanases that can be used at an alkaline pH and high temperature—conditions that are applied for pulp bleaching in pulp and paper industries [14]. Chemical chlorinated compounds can be substituted by enzymes (biobleaching) to reduce the environmental impact of the process as well as to reduce cellulose damage. Moreover, biobleaching enhances the paper quality and minimises the production cost of paper [15]. Thermostable xylanases of fungal origin are widely used in the bakery and food processing industries. The hydrolytic activity of these enzymes towards hemicellulose in wheat flour leads to a softer dough, thus increasing the volume of the bread. In addition, xylanases can be used (i) to separate wheat starch and gluten more easily [16], (ii) to improve the texture and tastiness of cream crackers [17], and (iii) to produce dextran(s), which is widely used as a food thickener [18]. The soft drink industry can also take advantage of the use of xylanases, in combination with pectinases and cellulases, to reduce fruit juices’ turbidity and viscosity. This improves pulp liquefaction and, in turn, juice organoleptic features. In addition to food production, xylanases are also used to enhance the quality of animal feed; for instance, they are used to improve the digestibility of agricultural silage, which is used to feed livestock [14]. Xylanases have been also reported to aid biofuel production because they significantly improve the saccharification of LCB [19,20,21].

In LCB, xylan polymers cover the surface of cellulose fibres, blocking the access of cellulases to their substrate. Therefore, xylanase activity has a double function: (i) the release of pentose sugars from hemicellulose and (ii) rendering cellulose more available by increasing fibre swelling and porosity. Hydrolysis of LCB releases both hexose (C6) and pentose (C5) sugars, increasing the yield of fermentable sugars. For instance, Choudhary et al. (2014) [22] have reported that the addition of xylanases to cellulases during the saccharification process resulted in an increase (of about 69.5%) in the release of sugars (glucose and xylose) from the biomass [19] (Bajaj and Mahajan, 2019). However, exploitation of LCB is challenging due to the high complexity and recalcitrance of enzymatic hydrolysis. Therefore, the discovery of robust hydrolytic enzymes represents a key factor in setting up a more effective biomass deconstruction process and, in turn, aiding circular economy-inspired industrial applications. In this framework, the implementation of thermophilic microbes [7,23,24] and enzymes [25] in biorefineries can lead to several advantages, like the increase in (i) reaction rates, (ii) substrate and product solubility, and (iii) diffusion coefficient [7]. Nevertheless, lignocellulolytic systems of single microbial strains often lack one or more key hydrolytic enzyme(s), which results in the inefficient breakdown of lignocellulose. Although several microbial strains have been found to be involved in lignocellulose deconstruction in several natural environments, the low cultivation efficiency of these strains in controlled laboratory conditions, as well as the costs related to the heterologous production of their hydrolytic enzymes, can be considered some of the major drawbacks for the development of a low-cost biorefinery technology [26,27].

An alternative and effective approach to overcome this downside is the use of deconstructing microbial consortia (also known as microbiomes) [28,29,30]. Indeed, microbiomes from different natural ecosystems exhibit highly efficient lignocellulose degradation due to the combined and synergic action of multiple enzymes produced by microbes from diverse taxonomic groups [31]. In addition to natural environments, some industrial ecosystems (like anaerobic digestors) hold great potential as a source of microbes and enzymes for the hydrolysis of lignocellulose. Metagenomic analyses of ecosystems populated by multispecies consortia can help in studying microbial diversity as well as identifying the lignocellulolytic enzymes involved in the deconstruction of lignocellulose [32,33,34]. Moreover, this approach offers the advantage of uncovering genes that encode enzymes with unique properties, such as enhanced tolerance to salt, high temperature, and ionic liquid. Nevertheless, directly identifying microorganisms and enzymes involved in lignocellulose deconstruction within complex ecosystems can be challenging. To overcome this, enrichment cultures can be employed by utilizing lignocellulosic biomaterial as the sole carbon source. This method generates microbial communities with an improved capacity to break down a specific LCB of interest. Although these enriched cultures cannot always be directly applied to controlled industrial degradation processes due to their dynamic and complex nature, they serve as a valuable resource for creating less complex and more efficient microbiomes (i.e., synthetic microbial consortia or co-cultures) for specific industrial applications as well as identifying the hydrolytic enzymes involved in the degradation process. 

Despite the extensive research conducted on CAZyme discovery and digestive microbiomes, there has been a noticeable lack of studies examining the utilization of anaerobic digestors as microbial inoculants for conducting enrichment cultures on SMS. Consequently, this study was devised and executed to investigate the diversity of microorganisms and CAZymes, while exploring the impact of different temperatures (37, 50, and 70 °C) and selective substrates (i.e., carboxymethylcellulose and xylan). Moreover, sub-enriched microbiomes were tested for the ability to produce hydrolytic enzymes in a cheap medium (i.e., a mixture of SMS and digestate). Secreted enzymes (i.e., secretomes) were characterised in terms of temperature and pH optima, thermal stability (including shelf-life at 4 °C), and types of products released upon hydrolysis of pure hemicellulose-derived polymers. 

## 2. Results

### 2.1. Time Course Analyses of the Enrichment Cultures

**Figure 1:**
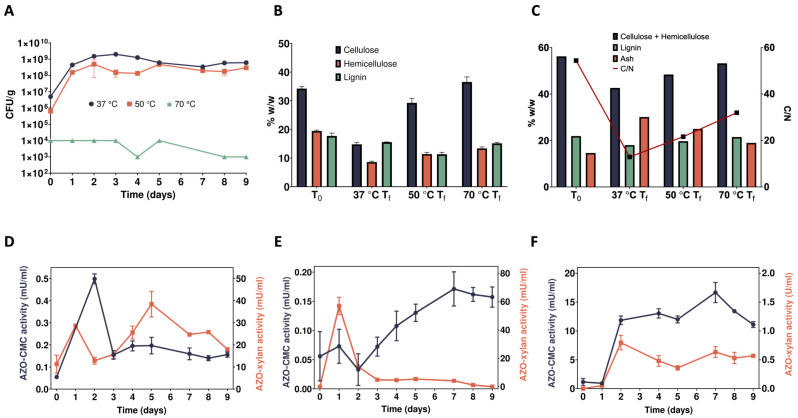
Time course analyses of the enrichment cultures. (**A**) Microbial biomass; (**B**) WIS composition, as determined by TCA; (**C**) WIS composition, as determined by thermal analysis, and C/N ratio; (**D**–**F**) detection of endo-1,4-β-D-xylanase and endo-1,4-β-D-glucanase activities in the culture supernatants of the enrichment cultures at 37, 50, and 70 °C, respectively.

SMS was used as the sole carbon source in enrichment cultures to select for lignocellulolytic microbial consortia (also known as microbiomes) able to break down this abundant agricultural leftover. Three temperature regimes were applied (i.e., 37, 50, and 70 °C) to evaluate the effect of temperature on the selection process of lignocellulose-degrading microbes. Enrichment cultures were sampled daily to monitor microbial proliferation, as well as endo-1,4-β-D-xylanase and endo-1,4-β-D-glucanase activities in the culture supernatant, and the residual content of cellulose, hemicellulose, and lignin; elemental (CHNS) and thermal (TG-DSC) analyses were carried out at T_0_ as well as at the end of the enrichment cultures (T_f_). Colony-forming unit (CFU) counting was performed to measure microbial proliferation as a consequence of SMS utilization as a carbon source (Figure 1A). Despite the use of the same amount of microbial inoculum (i.e., digestate), at 37 °C, the initial microbial biomass was the highest with almost 10^7^ CFU per gram of water-insoluble solid (WIS) (CFU/g_WIS_), followed by the enrichment cultures at 50 °C (about 10^6^ CFU/g_WIS_) and 70 °C (around 10^4^ CFU/g_WIS_). This likely depends upon the major fraction of mesophilic microbes in the digestate rather than the thermophilic counterparts. Microbial biomass increased from 10^6^ to about 10^8^–10^9^ CFU/g_WIS_ over the first 2 days of cultivation at 37 and 50 °C, whereas later, the biomass was stably maintained. Conversely, at 70 °C, the microbial biomass was rather stable, with slight fluctuations around 10^3^–10^4^ CFU/g_WIS_ throughout the whole enrichment period (Figure 1A). These results correlate well with the fact that the initial microbial inoculum comes from a biogas plant that is operated under mesophilic conditions. However, although the microbial biomass was not increasing over time, it is worth noting that microbial proliferation was detected even at a temperature of 70 °C when performing CFU estimation (Figure 1A).

Regarding SMS utilization, compared to the initial content (dry weight basis) of cellulose (34.2% *w*/*w*), hemicellulose (19.4% *w*/*w*), and lignin (17.7% *w*/*w*) (Figure 1B, T_0_), all polymers were in part consumed by the microbiome selected at 37 °C. In fact, after 9 days of cultivation, the content of cellulose, hemicellulose, and lignin decreased to 14.8% and 15.6%, respectively (Figure 1B, 37 °C T_f_). As for the enrichment culture at 50 °C, this microbiome preferred hemicellulose and lignin as carbon sources. Indeed, it only managed to reduce the cellulose content from 34.2 to 29.3% *w*/*w*, whereas only about 11% of both hemicellulose and lignin was left at the end of the cultivation (Figure 1B, 50 °C T_f_). Similarly, but with weaker hydrolytic capacity, the microbiome enrichment at 70 °C preferred hemicellulose and lignin; indeed, it reduced their content down to 13.4 and 15.1%, respectively, whereas cellulose was apparently not hydrolysed at all (Figure 1B, 70 °C T_f_). This degradation trend is also confirmed by thermal analysis (Figure 1C; Table S1). Analysing peat and plant materials [35] found three main regions of weight loss (WL) in thermogravimetric curves; in particular, that occurring between 275 °C and 325 °C was associated with readily oxidizable compounds such as sugars and cellulosic materials, while that in the 360–460 °C region was linked to more recalcitrant structures including lignin. Using similar temperature ranges, confirmed also in this study (Figure S1) and previous studies on digestates [36], the content of cellulose + hemicellulose (WL_250–350_) and lignin (WL_350–450_) was estimated before the incubation (56.3 and 21.9%, respectively) at T_0_ as well as after the incubation at different temperatures (T_9_). In particular, at 37 °C, both cellulose + hemicellulose and lignin content were partially degraded, with their concentrations decreasing down to 42.6 and 18.0%, respectively, while the ash content increased from 14 to 30% (Figure 1C, 37 °C T_f_; Table S1). At 50 °C, the residual content of lignin (19.7%) was slightly lower than the initial one (21.9%), while that of cellulose + hemicellulose decreased, although at a lower extent compared to the incubation at 37 °C, i.e., from 56.3 to 48.4% (Figure 1C, 50 °C T_f_; Table S1). Just a very faint degradation occurred following microbiome enrichment at 70 °C; in fact, the content of cellulose + hemicellulose, lignin, and ash (53.2, 21.5, and 19.0%, respectively) was like that measured at T_0_ (Figure 1C, T_0_ and 70 °C T_f_; Table S1). The hydrolytic activity and/or the growth of the microbial community are mirrored by the C/N ratio, which showed the lowest value (13) at 37 °C and the highest at 70 °C (32) (Figure 1C). It is worth noting that, while concentrations of cellulose + hemicellulose obtained using different techniques may appear diverse, they are positively correlated (*p* < 0.01). Conversely, no correlation was found for lignin, even though in both cases the pattern was the same.

To confirm the presence of microorganisms secreting lignocellulolytic enzymes, cell-free supernatants were sampled daily from the enrichment cultures and tested using the chromogenic substrates Azo-Xylan and Azo-CMC (Figure 1D–F). The supernatant of the microbiome selected at 37 °C showed a sharp increase in the Azo-CMCase activity (up to about 500 μU/mL) within 2 days of cultivation, which then stabilised at around 100–200 μU/mL. On the other hand, the Azo-Xylanase activity was, overall, two orders of magnitude higher (mU/mL) than the Azo-CMCase activity (μU/mL) and showed a gradual increase until the fifth day of cultivation (up to about 40 mU/mL). Later, it slowly declined down to 15 mU/mL (Figure 1D). An opposite trend was detected for the supernatant of the microbiome selected at 50 °C. In this case, the Azo-Xylanase activity topped its value around 60 mU/mL within 1 day of cultivation, whereas the Azo-CMCase activity, aside from a slight initial drop, gradually increased from the second to the seventh day of enrichment (i.e., from about 50 to 150 μU/mL), then it stabilised for a couple of days around 150 μU/mL, before eventually declining to about 150 μU/mL (Figure 1E). Regarding the microbiome selected at 70 °C, both Azo-CMCase and Azo-Xylanase activities gradually increased until the seventh day of cultivation up to 15 mU/mL and 0.6 U/mL, respectively. Later, while the Azo-Xylanase activity stabilised, the Azo-CMCase counterparts declined to 10 mU/mL (Figure 1F). The higher activity of the secretome of the microbiome selected at 70 °C can be explained by a high affinity of the secreted enzymes for the Azo-Xylan substrate. On the other hand, the weaker microbial proliferation and degradation of the SMS during the enrichment can be ascribed to fewer microbes present in this sample that do not secrete a complete enzymatic arsenal for the degradation of the complex LCB. Although enzyme activities in the range of μU-mU per mL of supernatant seem rather low, it must be considered that the activity towards model substrates, like Azo-Xylan and Azo-CMC (Figure 1D–F), does not directly reflect the activity of these enzymes on their natural substrates but only proves their presence in the culture supernatants. Indeed, the decreased residual content of cellulose, hemicellulose, and lignin (Figure 1B) confirms the overall ability of these microbiomes to hydrolyse native lignocellulose. Therefore, PCR-DGGE and metagenomic analyses have been carried out to study the variation in microbial population composition because of the selection process (see Section 2.6) and to identify the families of carbohydrate-active enzymes (CAZymes) potentially involved in lignocellulose deconstruction (see Section 2.7).

### 2.2. Mesophilic Microbial Consortia Secrete Thermophilic and Thermostable Xylanases

Microbial consortia were isolated from the main enrichment cultures with the aim of selecting cellulolytic and hemicellulolytic subpopulations. Five sub-enriched consortia were isolated based on the three temperatures (37 °C, 50 °C, and 70 °C) and two carbon sources (CMC and xylan) (see Section 4.3). Sub-enriched consortia were proliferated in the presence of sterile SMS and digestate to induce lignocellulosic enzyme secretion. No soluble cellulases, also known as secretomes, were secreted into the culture supernatants. For this reason, we focused on the xylanase activity that was preliminarily measured via colourimetric Azo-Xylan assays. Given the stronger hemicellulose degradation of consortia by the secretomes sub-enriched at 37 °C, these were subjected to an in-depth characterization. 

**Figure 2:**
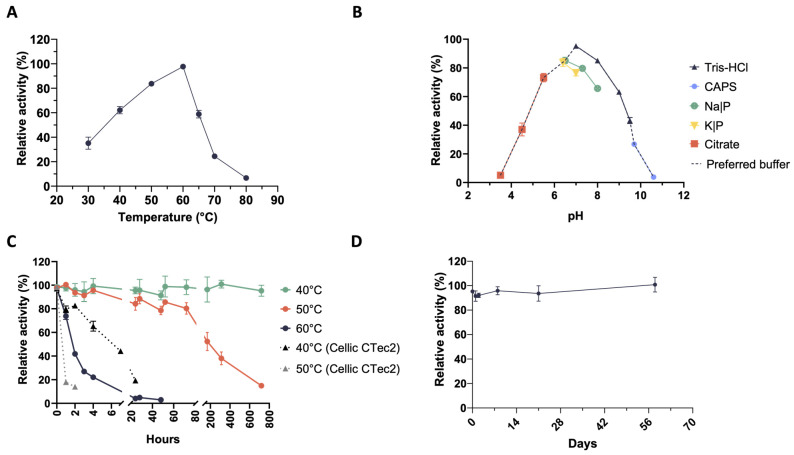
Biochemical characterisation of xylanase activities in the secretome from the microbiome CMC-37. (**A**) Temperature optimum; (**B**) pH optimum; (**C**) thermostability; (**D**) storage stability.

Temperature and pH are among the most important parameters influencing biocatalytic processes. Therefore, we have measured enzyme activity over the temperature range of 30–80 °C and the pH range of 3.5–10.5. Xylanases secreted by the microbial consortium (also known as the microbiome) CMC-37 are catalytically active from 30 °C to 70 °C, with a temperature optimum at 60 °C, whereas they almost completely lost activity at 80 °C (Figure 2A). 

Based on these results, the assays carried out to determine the pH optimum were run at 60 °C. Xylanases were active in the range of 5.5–9.5, with an optimum level of activity at pH 7. It was also shown that the enzymes were almost completely inactive at extremely acidic or alkaline conditions and that the degree of activity depended on the type of buffer used for the assay. Indeed, at the same pH value, enzymes performed better in the Tris-HCl buffer (Figure 2B), which was chosen for further analyses. The thermostability of the secretome was tested at 40 °C, 50 °C, and 60 °C. Interestingly, xylanases in the secretome kept 95% of their initial activity for up to 30 days (720 h) when incubated at 40 °C (Figure 2C), whereas at 50 °C they retained more than 80% of their activity for 72 h (3 days). Instead, the catalytic activity rapidly decreased at 60 °C until it was almost undetectable after 24 h. Xylanase activity dropped to 22% within 4 h and to 4% after 24 h (Figure 2C). To compare the stability of these xylanases with that of a commercially available enzyme mixture, we tested the loss of activity of Cellic^®^ CTec2 (0.04 FPU/mL in digestate) (Novozymes A/S) at 50 °C (its temperature optimum) as well as at 40 °C. A rapid activity drop (from 100% to 20%) was detected already after 1 h of incubation at 50 °C. Even if the Cellic^®^ CTec2 mixture was more stable at 40 °C, it completely lost activity within 24 h of incubation (Figure 2C). This instability was observed when the commercial mixture was tested in digestate to mimic the same environment of the enzymes present in the secretomes. On the other hand, when tested in enzyme assays using defined buffers, Cellic^®^ CTec2 outperformed (in terms of stability) the secretome from the microbiome CMC-37. To study the storage stability (i.e., shelf life) of the xylanases in the CMC-37 secretome, their stability was tested over time at storage temperature (4 °C). As it is shown in Figure 2D, no apparent loss of activity was detected over a period of about 2 months. 

**Figure 3:**
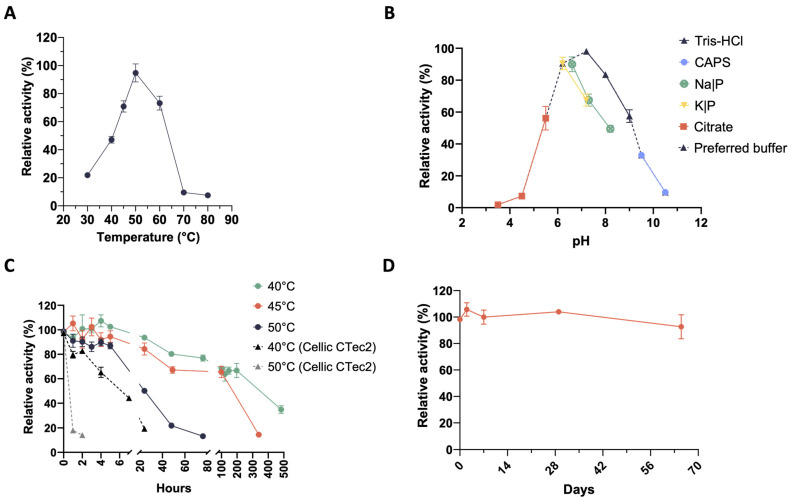
Biochemical characterization of xylanase activities in the secretome from the microbiome XYL-37. (**A**) Temperature optimum; (**B**) pH optimum; (**C**) thermostability; (**D**) storage stability.

With regard to the microbiome XYL-37, secreted xylanases are active over the temperature range of 30–60 °C with a temperature optimum at 50 °C. In this case, enzymes are slightly less thermophilic; indeed, they show a very low activity already at 70 °C (Figure 3A). Xylanases performed well in the pH range of 5.5–9.5; however, these enzymes seemed to be more easily inactivated in acidic conditions, i.e., already at pH 4.5. In fact, whereas those produced by CMC-37 retained 37% of their maximum activity at pH 4.5 (Figure 2B), those from XYL-37 only showed 7% of their maximum activity (Figure 3B). Moreover, the type of buffer strongly influenced enzyme activity even at the same pH value. For instance, at pH 7, xylanases were 30% less active in sodium phosphate than in Tris-HCl (Figure 3B), which again was used for further secretome characterisation. Thermostability was tested at their temperature optimum (50 °C) as well as 5 and 10 °C below it (Figure 3C). During the first 24 h, enzymes kept more than 80% of their initial activity at both 40 and 45 °C. Then, they slowly inactivated to about 65% of the initial activity between 100 and 200 h of incubation. Instead, at their temperature optimum (50 °C), enzymes kept more than 80% of their activity for up to 5 h. Then, they rather quickly lost activity within 76 h of incubation. Also, in this case, the secreted enzymes were found to be more thermostable than those of the Cellic^®^ CTec2 mixture (Figure 3C). 

Regarding their shelf life, there was no evident decrease in activity over a time span of approximately 2 months (Figure 3D). Considering that these enzymes have not been purified from the digestate, which is a very complex matrix, this is an encouraging result. Indeed, it is expected that these biocatalysts could be even more stable if included in a formulated enzyme mixture and could be stored for a long time before being used in the desired bioprocess. Moreover, despite being produced by microbes selected at 37 °C (mesophiles), these xylanases show temperature optima (50 °C and 60 °C) very similar to that of enzyme cocktails that are used in second-generation biorefineries for biomass saccharification. Therefore, these enzymes could be used to complement these commercial cocktails to improve the hydrolysis of hemicelluloses.

### 2.3. Electrophoretic and Zymographic Analyses 

**Figure 4:**
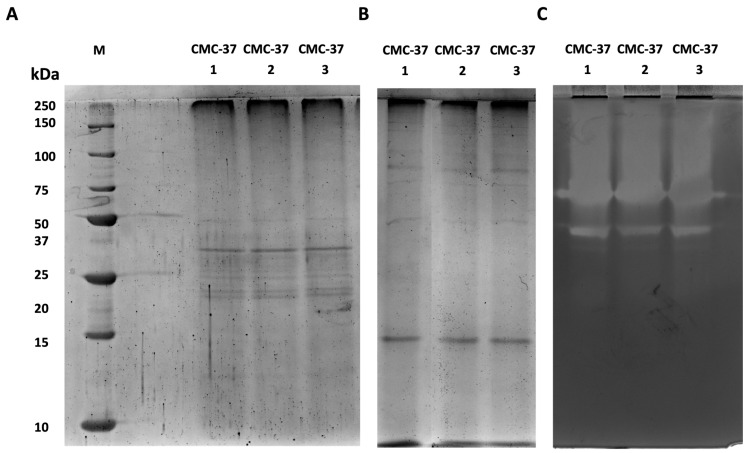
Electrophoretic and zymographic analyses of three independent secretomes from the microbiome CMC-37. (**A**) SDS-PAGE; (**B**) native PAGE; (**C**) zymogram. M: Precision Plus Protein Standard Unstained (Bio-Rad).

One step towards the identification of specific enzymes present in the secretomes is their visualisation via electrophoretic analysis as well as the detection of their in-gel activity by zymography. To do so, we have run electrophoretic separation in both denaturing and native conditions. Given the heterogeneity of the isolated microbiomes, these analyses were run on three independent samples to evaluate whether enzyme production was consistent. As is shown in Figure 4A, secretome samples from the CMC-37 microbiome showed the same pattern of protein bands with the most abundant proteins showing molecular weights between 20 and 50 kDa. Less abundant proteins, with higher or lower molecular weights, were also visible but as very faint protein bands (Figure 4A). Native SDS-PAGE (Figure 4B) followed by zymography (Figure 4C) revealed the presence of three major distinct bands of xylanase activity. However, given the presence of an activity smear at the topside of the gel, the presence of other xylanases could not be excluded.

**Figure 5:**
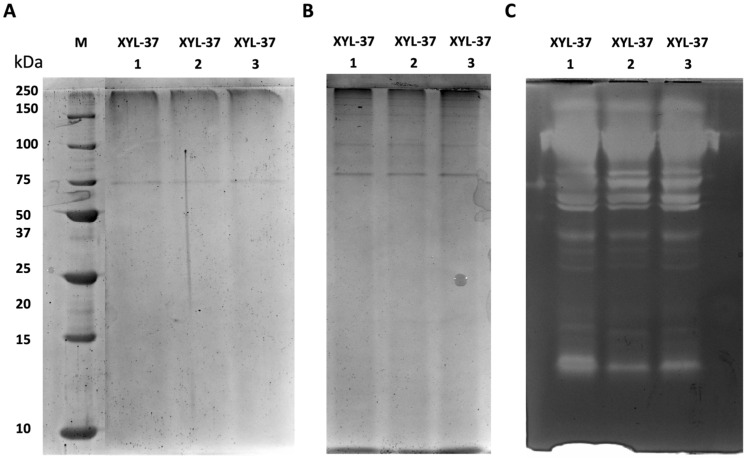
Electrophoretic and zymographic analyses of three independent secretomes from the microbiome XYL-37. (**A**) SDS-PAGE; (**B**) native PAGE; (**C**) zymogram. M: Precision Plus Protein Standard Unstained (Bio-Rad).

Regarding enzymes in the secretome of the XYL-37 microbiome, the results from three independent replicates were consistent with each other. However, in this case, fewer protein bands were visible in both denaturing and native SDS-PAGE (Figure 5A,B). Nonetheless, many activity bands were detected in the zymogram (Figure 5C); this indicates that, despite enzymes being catalytically active, their amount falls below the limit of detection of the Coomassie staining. It is worth noting that the zymography revealed the presence of at least 13 distinct bands (Figure 5C), pointing to the presence of a higher number of distinct xylanases compared with those present in the secretome of CMC-37. 

### 2.4. Determination of Enzyme Units 

**Figure 6:**
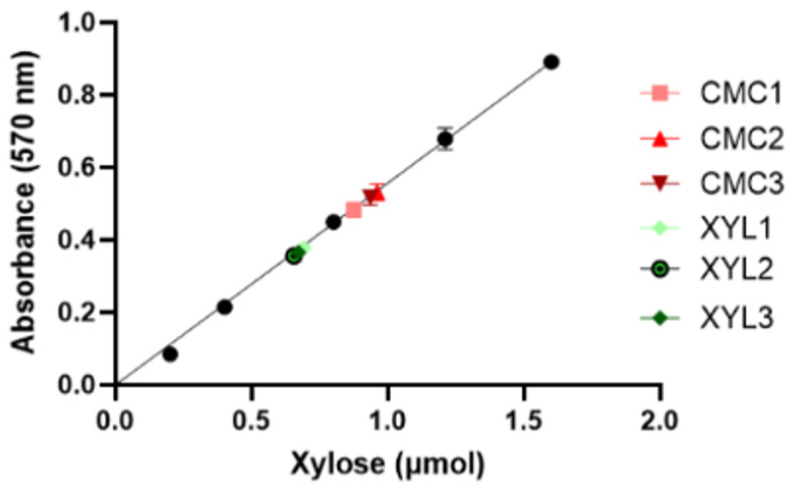
Calibration curve for the determination of the amount of released xylose. Curve equation is Y = 0.5571 × X, the R^2^ is 0.9995.

The enzyme’s catalytic activity of the secretome, expressed as enzyme units per mL (U/mL), was measured by means of the DNS assay using pure xylan as the substrate. One unit (U) of xylanase is defined as the amount of enzyme required to release one micromole (μmol) of xylose in a minute (1U = 1 μmol/min). First, a calibration curve was obtained by carrying out the DNS assay on standard samples containing a known amount of xylose (Figure 6). Then, enzyme reactions were started by mixing the same volumes (35 µL) of culture supernatant and 1% *w*/*v* xylan solution. The reactions were incubated at the optimum conditions of temperature and pH before adding the DNS reagent and measuring the absorbance at 570 nm (see Section 4.4 in Material and Methods). The incubation time was 10 or 30 min for the supernatants of the CMC-37 and XYL-37 supernatants, respectively. Based on the absorbance of the unknown samples, the calibration curve was used to calculate the amount of xylose (in µmol) released during the enzyme assay (Figure 6).

By considering the volume of supernatant used for the assay and the incubation time, the catalytic activity values (U/mL) of all the secretomes tested are summarised below in Table 1.

This is an encouraging result considering that, for enzyme production, the two microbiomes were cultured in an inducing medium made of two industrial leftovers (i.e., wheat straw litter + digestate), without supplementation of any additional nutrient. Therefore, it can be expected that higher amounts of enzymes can be produced by optimizing the production process and media formulation.

### 2.5. LCB-Derived Polysaccharides Hydrolysis Products Profiling 

**Figure 7:**
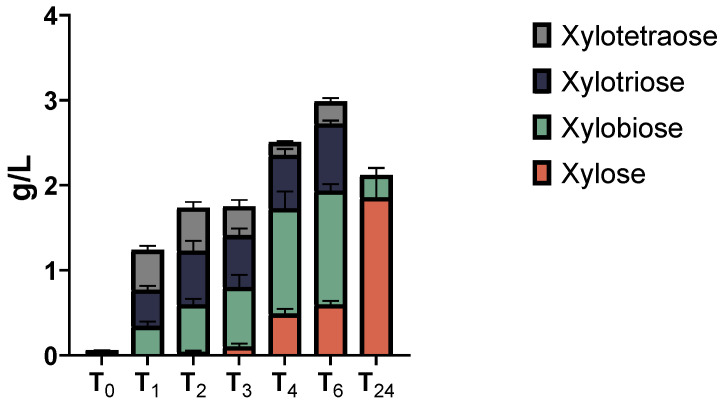
Time course of xylan degradation products obtained using the secretome of the consortium XYL-37. Each bar represents the amount of xylose (red), xylobiose (green), xylotriose (blue), and xylotetraose (grey) released after 0, 1, 2, 3, 4, 6, and 24 h of digestion.

To obtain information about the type of secreted xylanases, the secretome of the XYL-37 microbiome was used to hydrolyse pure beechwood xylan, and the reaction products were analysed via HPAEC-PAD. A time-course analysis was carried out by stopping the hydrolytic reactions at 1, 2, 3, 4, 6, and 24 h post-enzyme addition. As is evident from Figure 7 and Figure S2, and Table S2, in addition to xylose, oligos with different degrees of polymerization (DP) are released from the polymeric substrate. These oligos show a retention time of about 10.2, 13.2, and 17.0 min, and compared with standards (Megazyme) it was found that they are dimers (i.e., xylobiose), trimers (i.e., xylotriose), and tetramers (i.e., xylotetraose) (Figure S2). The product profile shown in Figure 7 confirms the presence of both endo-1,4-β-D-xylanases and exo-1,4-β-D-xylosidases in the secretome. Judging from the height of the peaks (Figure S2) and the quantitative data (Figure 7), at the beginning of the reaction (up to 2 h of incubation), an increased amount of all oligos was measured (Figure 7; 0–2 h); this is likely due to the activity of endo-1,4-β-D-xylanases. Later, the concentration of the two shorter products (DP2 and DP3) increases at the expense of the longer one (DP4), and eventually, they were all converted to xylobiose and xylose due to the activity of exo-1,4-β-D-xylanases (Figure 7; 24 h).

**Figure 8:**
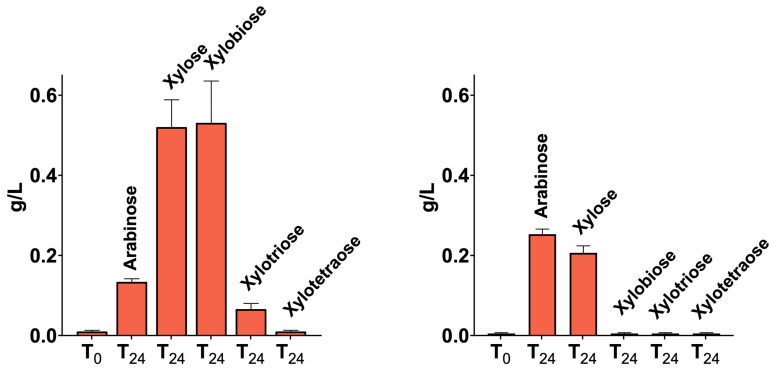
Arabinoxylan degradation products obtained using the secretomes of the consortia CMC-37 (**A**) and XYL-37 (**B**). Each bar represents the amount of arabinose, xylose, xylobiose, xylotriose, and xylotetraose released after 0 and 24 h of digestion.

The secretomes of CMC-37 and XYL-37 microbiomes were tested also for the hydrolysis of wheat flour arabinoxylan (P-WAXYL, Megazyme), tamarind xyloglucan (P-XYGLN, Megazyme), and ivory nut mannan (P-MANIV, Megazyme), and the products were analysed via HPAEC-PAD. No activity was measurable towards tamarind xyloglucan, which is in line with the absence of cellulolytic activity in the secretomes. Instead, both secretomes were active towards wheat flour arabinoxylan (Figure S3) and produced xylose, xylooligosaccharides, and arabinose (Figure 8), thus pointing to the presence of α-L-arabinofuranosidases. Moreover, only the secretome of the XYL-37 microbiome was able to release oligosaccharides from ivory nut mannan (Figure S3).

### 2.6. Microbial Consortia Composition through PCR-DGGE and Metagenomic Analyses

**Figure 9:**
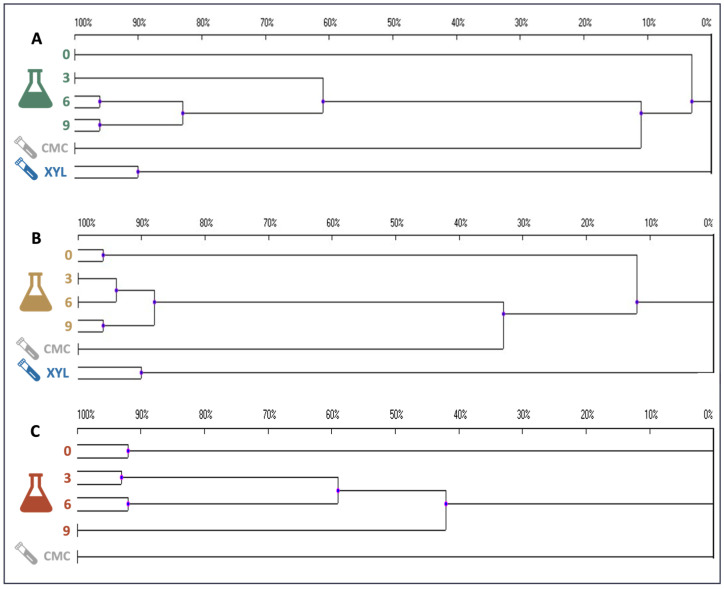
Dendrograms showing the similarity indices of the different DGGE profiles in enrichment cultures carried out at (**A**) 37, (**B**) 50, and (**C**) 70 °C.

DGGE profiling of the enrichment cultures was carried out to preliminarily assess variations in the composition of the microbiomes as a consequence of the temperature and substrate constraints applied (Figure S4). The pattern of amplification bands suggests the presence of numerous bacterial species in the initial sample. To quantitatively compare the similarity of the DGGE amplification patterns, a statistical analysis was carried out and dendrograms were constructed (Figure 9). For all the enrichment cultures, a drastic variation in the composition of the microbiome was rapidly evident; indeed, already after 3 days of cultivation on SMS, the microbial populations displayed either very low (about 13%) or no similarity compared to the initial inoculum profile (Figure 9). Later, at 37 and 50 °C, the microbiome tended to stabilise, as suggested by the overtime increase in profile similarity (Figure 9); this is likely due to the establishment of bacterial species able to feed on SMS at the specific temperature of selection. On the other hand, with regard to the enrichment culture incubated at 70 °C, the microbiome was more variable over time. As evident from the very weak ability to degrade SMS (Figure 1B), it is likely that the selection of lignocellulolytic microbes was unsuccessful at this high temperature. However, the predominance of amplification bands in the lower part of the gel points to the proliferation of thermophilic bacteria that tend to have a higher percentage of GC in the genome [37]. DGGE profiling was carried out also on samples that were further selected on model substrates (i.e., CMC and xylan), as described in Section 4.3. Microbiomes selected on CMC at 37 °C, 50 °C, and 70 °C exhibited profile similarity of about 11, 35, and 0% with populations selected on SMS at the same temperature. No microbial proliferation was observed on pure xylan at 70 °C, whereas samples selected at 37 °C and 50 °C were single clusters showing high diversity when compared to all other samples (Figure 9).

To gain a clearer picture of the microbial communities’ composition in the selected microbiomes, a total of 285,917,793 150 bp paired-end reads were generated for the nine microbiomes, with an average read count of about 31 M per sample. Data on the metagenome of the sample XYL-70 are not reported because no microbial proliferation was observed on a solid medium containing xylan as the sole carbon source. More than 99% of the reads passed the preprocessing step (quality trimming and contaminant removal). At first, we performed a taxonomy classification at the read level using kraken2 against the GTDB database to gather a general overview of the microbial composition (Figure S5). As expected, the initial sample (T_0_-SMS) and the three microbiomes, selected at 37 °C, 50 °C, and 70 °C, contain the higher variability in terms of identified phyla. On the other hand, the microbiomes sub-enriched on carboxymethylcellulose (i.e., CMC) or corncob xylan (i.e., XYL) are dominated by just one phylum. The microbiomes CMC-37 and XYL-37 are mainly composed of proteobacteria, while firmicutes dominate in microbiomes enriched on CMC or XYL at 50 °C and 70 °C (Figure S5).

**Figure 10:**
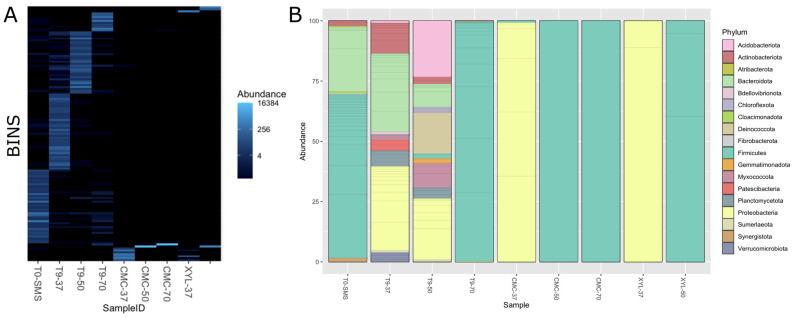
Panel (**A**): bins distribution across the different samples. Abundance values are expressed as “genome copies per million reads”, as calculated with Salmon implemented in the metaWRAP pipeline. Panel (**B**): bins’ taxonomic classification based on GTDB database. Abundance values are expressed as fraction of the total count.

**Figure 11:**
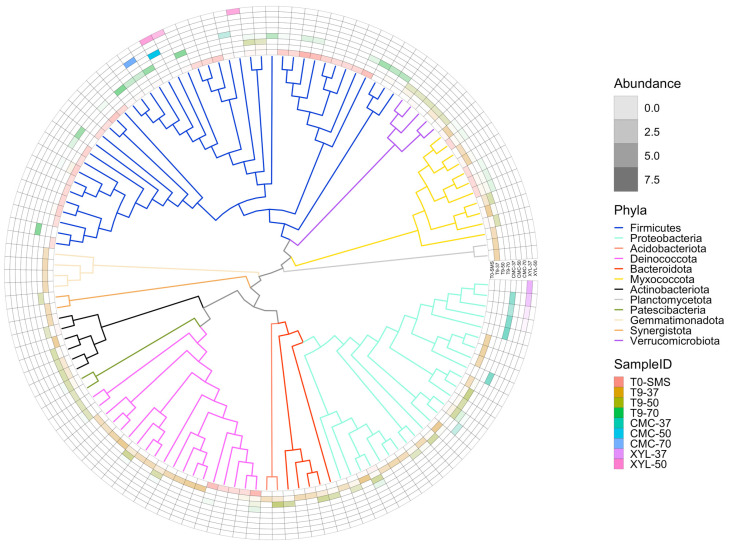
Bin phylogenetic tree based on GTDB database. To make the figure more readable, only the bins belonging to phyla represented more than once are reported (125 bins out of 131). The outer circle represents the bin abundance in a specific sample expressed as “genome copies per million reads”. The abundance values are log-scaled.

Metagenome assembly was performed independently for the different microbiomes. Assembly metrics show a wide variability among the datasets, reflecting the different composition and complexity of the microbiomes (Table 2). Samples T_0_-SMS and T_9_-37 contain the higher number of contigs and the lowest N50, 52,084 (N50 3538) and 65,657 (N50 6133), respectively, while sample CMC-70 has the smallest contig number, with only 37, and an N50 higher than 360 Kb. The binning steps were performed using three different binning algorithms that were further improved by the binning tool implemented in MetaWRAP. We only retain metagenome-assembled genomes (MAGs) that have a completeness of at least 50% and a contamination frequency lower than 5% for a total of 132 bins. A higher number of MAGs were retrieved in microbiomes T_9_-37 and T_0_-SMS, with 39 and 38 MAGs, respectively, while microbiomes selected on CMC and XYL contain the lowest number of MAGs (Table 3). To obtain a final list of nonredundant MAGs across all microbiomes, we performed a dereplication step that produced a final number of 131 MAGs (only one MAG in the microbiome XYL-50 matches with a MAG in the microbiome CMC-50). The full list of quality metrics calculated with checM is available (Table S3). As shown in Figure 10A, the microbial composition is quite different among samples with few overlaps, indicating that the dominant species (i.e., the ones that have sufficient coverage and have been assembled into bins) change because of the different cultivation conditions applied. The impact of the selection (temperature and/or polymeric substrate) is also evident considering the alfa diversity, which is a measure of microbiome complexity (Figure S6). In T_0_-SMS and T_9_-37, we observed a higher microbial diversity that tended to decrease as a function of the increasing temperature and substrate selection. MAGs’ taxonomic classification is in line with what we observed using kraken2 at the read level. By considering the distribution of the bins across the samples (Figure 10A), it is evident that the various samples exhibit significant dissimilarities and possess distinct microbiome compositions. In particular, the most abundant phyla in the T_0_-SMS sample are *Firmicutes* and *Becteroidota*, in the T_9_-37 sample are *Proteobacteria* and *Bacteroidota*, and in the T_9_-50 sample are *Proteobacteria*, *Acidobacteriota* and *Deinococcota* (Figure 10B). Considering the other samples, we observed that those sub-enriched at 50 and 70 °C contain almost only *Firmicutes* (sample CMC-50 contains only one MAG classified as *Bacillus licheniformis*, while sample CMC-70 has one MAG annotated as *Parageobacillus thermoglucosidasius*), while microbiomes sub-enriched at 37 °C are exclusively composed of *Proteobacteria* (Figure 10B and Figure 11).

### 2.7. Meta-Functional Analysis of CAZymes

The metagenomics functional profile of the enriched microbiomes was explored through the annotation of the carbohydrate-active enzymes (CAZymes) with the tool Search with dbCAN2 HMMs. As such, a total of 9925 putative CAZyme sequences were inferred (Table 4) and assigned to 239 families.

**Figure 12:**
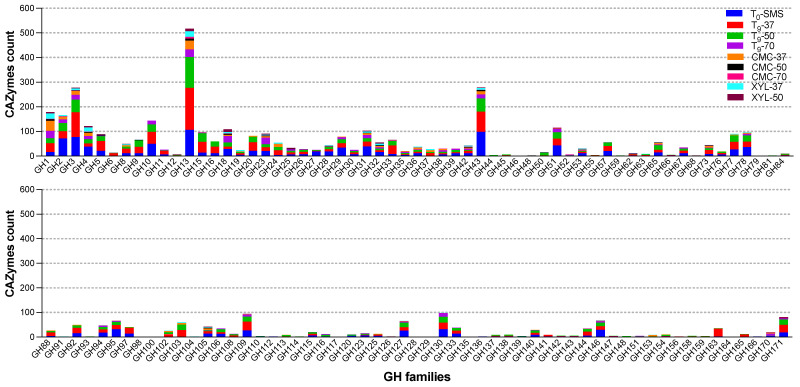
Relative abundance of the GH families across all the metagenomes.

All detected open reading frames (ORFs) were further grouped into six different functional classes: GHs, GTs, CEs, AAs, PLs, and CBMs. The majority of all the CAZyme ORFs (49.5%) were represented by GHs, whereas the PLs (2.0%) and CBMs (2.2%) were very scanty in the community. The relative abundance of the other classes was 32.4% (GTs), 10.0% (CEs), and 3.9% (AAs), respectively. GHs are known for their prominent hydrolysing activity towards carbohydrate substrates, such as lignocellulose, starch, and chitin. In total, 4914 ORFs belonging to 116 different GH classes (Figure 12) were detected. It is noteworthy that the highest number of GHs (1477 ORFs) is also in line with the number of MAGs (39) identified in the metagenome of the microbiome T_9_-37 (Table 2 and Table 3), whereas due to low microbial diversity, the sub-enriched samples CMC-50 (*Bacillus licheniformis*) and CMC-70 (*Parageobacillus thermoglucosidasius*) possess the lowest number of ORFs coding for GHs identified (58 and 32, respectively).

Within GHs, the most abundant family across all nine microbiomes were GH13 (517 ORFs), of which 171 and 125 ORFs were found in T_9_-37 (in red) and T_9_-50 (in green), respectively (Figure 10 and Table 4). This family, together with GH4, GH31, GH57, GH63, GH97, and GH126, can be linked to starch degradation by encompassing starch- and pullulan-modifying enzymes, including α-amylases, pullulanases, α-1,6-glucosidases, neopullulanases, and cyclodextrinases [38]. From a biotechnological point of view, amylases are among the most important enzymes for the food industry and recently have found applications also in biofilm inhibition [39].

**Figure 13:**
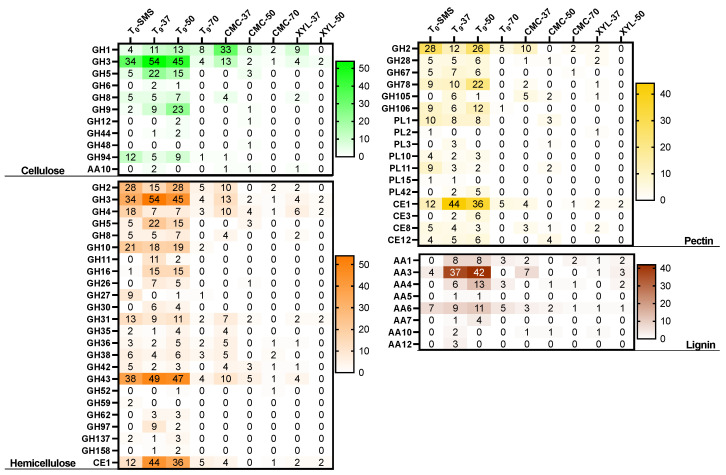
Heatmap of CAZy functional terms. A comparison of the potential capacities of each microbiome in degrading lignocellulosic polymers (i.e., cellulose, hemicellulose, pectin, lignin). Abbreviations: glycoside hydrolase (GH), glycosyl transferase (GT), polysaccharide lyase (PL), carbohydrate esterase (CE) and carbohydrate-binding module (CBM).

To reveal the metabolic potential for lignocellulose biomass transformation, ORFs encoding for cellulose-, hemicellulose-, pectin-, and lignin-degrading enzymes were identified across the nine microbiomes (Figure 13). A total of 11 families were linked to cellulose degradation. In particular, the oligosaccharide-degrading enzymes (GH1, GH3, and GH94) were the most dominating in all the microbiomes. Several classes of endo-glucanases and/or cellulases were identified (i.e., GH5, GH6, GH8, GH9, GH44, and GH48). After 9 days of the enrichment cultivation, GH5, GH6, GH8, GH9, and GH44 members were found in all the microbiomes, except for T_9_-70 in which GH8 and GH44 were not found. This result suggests that this microbiome, represented by *Firmicutes*, acts on the internal cleavage of the cellulose backbone preferentially using specific hydrolases such as GH5, GH6, and GH9. Among the microbiomes enriched on CMC, the sample enriched at 37 °C showed the highest cellulose-degrading potential by accounting for 64 ORFs (out of a total of 280, Table 4), compared to CMC-50 and CMC-70 with only 15 and 3 ORFs, respectively. Indeed, the microbiome CMC-37 contains seven MAGs (six *Proteobacteria* and one *Firmicute*), whereas CMC-50 and CMC-70 are composed of unique MAGs, both belonging to *Firmicutes*. A similar microbial phylogeny and degradative potential are observed for (i) the microbiome XYL-37, represented by two strictly related species of *Klebsiella* (i.e., *K. oxytoca* and *K. pneumoniae*), belonging to *Proteobacteria*, and (ii) the microbiome XYL-50, in which three MAGs have been identified to belong to *Firmicutes* (*Fictibacillus gelatini*, *Brevibacillus borstelensis*, and *Bacillus licheniformis*). Interestingly, another class of CAZymes AA10 (formerly known as CBM33) has been annotated in five metagenomes: T_0_-SMS, T_9_-37, CMC-37, CMC-50, and XYL-37. Based on sequence similarity, the family AA10 contains enzymes known as lytic polysaccharide monooxygenases (LPMOs) that are active on cellulose, various types of hemicelluloses, chitin, starch, and/or oligosaccharides. 

A relevant number of 22 CAZy families (21 GHs and 1 CE) were identified to be related to hemicellulose degradation, with the highest number of 572 ORFs identified in sample T_9_-37. As for hemicellulose degradation, several oligosaccharide-degrading enzymes were annotated (i.e., GH2, GH3, and GH4) and the endo-xylanase activities fall as members of GH5, GH8, GH10, GH30, GH43, and CE1. GH3 and GH43 are the most represented families within 278 and 279 ORFs annotated, respectively. The former carries out a range of functions including α-L-arabinofuranosidase and β-D-xylosidase activities, whereas the latter mainly displays xylan degradation and bacterial cell wall remodelling. Also, in the context of hemicellulose degradation, the microbiome XYL-37 (most abundant microbes were *Klebsiella oxytoca* and *Klebsiella pneumoniae*) has shown a higher degradative potential compared to XYL-50 (Table 4 and Figure 13). It is reported that the strain THLC0409 of *Klebsiella oxytoca* was able to metabolise crystalline cellulose as well as natural substrates such as corncob, bamboo, and rice straw for ethanol production [40].

Pectin is an extremely complex polysaccharide, constituted by 17 different monosaccharides and more than 20 different kinds of linkages. In the analysed samples, 1110 ORFs encoding for putative pectin-degrading enzymes were annotated and are potentially acting through depolymerization (hydrolases and lyases) and/or de-esterification (esterases) reactions. Among these, 62.8% are represented by GHs, whereas 28.1% and 9.1% are belonging to CEs and PLs, respectively. Several CE8 and CE12 implicated in the removal of methyl and acetyl groups from pectin were annotated except in CMC-70, together with a few acetyl xylan esterases (CE3). This latter class was not identified when the selective growth on CMC and xylan was performed. The most abundant family was represented by feruloyl esterases (CE1) with a total of 231 genes identified in all the metagenomes, except in CMC-50, which was only composed of one bin assigned to *Bacillus licheniformis*. Other glycoside hydrolases (GH2, GH28, GH67, GH78, GH105, and GH106) are implicated in pectin degradation, together with PLs (PL1, PL2, PL3, PL10, PL11, PL14, and PL42). However, PLs were not found in CMC-37 and CMC-70, whereas six genes were identified in the bin *Bacillus licheniformis* (CMC-50). The same trend was observed also in the communities enriched on xylan. 

In comparison to the sugar-based components of the lignocellulose, lignin deconstruction is less understood. Therefore, eight AA members were identified, among which AA3 was the most abundant with 157 genes. This latter family comprises enzymes from the glucose–methanol–choline family of oxidoreductases involved in the reduction of toxic compounds such as quinones and radicals emerging from lignin degradation. Interestingly, in the sample T_9_-37, three members belonging to AA12 were identified, encoding for pyrroloquinoline quinone-dependent oxidoreductases, whereas the 1,4-benzoquinone reductases (AA6) are widespread in all the analysed metagenomes. Also, the AA4 and AA5 families were identified and are potentially active toward a variety of aromatic compounds produced during the degradation of lignin through enzymatic deconstruction. This process involves the well-studied laccases, belonging to the family AA1. In this study, 50 genes were annotated as AA1 and are mainly present in all the samples, except for CMC-50. From this analysis, like the other polymers, XYL-37 played a crucial role in the lignin deconstruction with 10 genes annotated as AAs; among those were 4 putative genes encoding for laccases. 

Similarly to cellulose, hemicellulose, and pectin, the pattern of CAZymes involved in lignin degradation is drastically reduced when the microbiomes have been exposed to selective high temperatures (i.e., 50 °C and 70 °C) as well as to pure polymeric carbon sources (i.e., xylan and CMC). These findings suggest that several factors might shape the microbiomes over time, driving the microbial compositions and, in turn, the degradative potential of the microbial communities.

## 3. Discussion

Lignocellulose biomasses (LCB), such as SMS, if not properly disposed of, can contribute to environmental pollution. LCB renewable resources can be used to produce biofuels (e.g., bioethanol, biogas, biohydrogen), chemicals (e.g., volatile fatty acids), and other value-added products (e.g., carbohydrate-active enzymes). The worldwide rise of the mushroom market is expected to be coupled with an increase in SMS that needs to be disposed of and/or valorised. Due to its substantial cellulose and hemicellulose content (approximately 57% on a dry weight basis), a potential biotechnological use of SMS involves utilizing it as a substrate for cultivating microbes in solid-state fermentation (SSF) or submerged fermentation (SmF); these processes aim to produce industrially relevant carbohydrate-active enzymes (CAZymes). Despite significant research into the discovery of CAZymes and digestive microbiomes, there has been a notable lack of studies exploring the use of anaerobic digesters as microbial sources to enrich cultures targeting SMS. Consequently, this study was designed and carried out to assess the microbial diversity and CAZymes while investigating how different temperatures (37 °C, 50 °C, and 70 °C) and substrates (like carboxymethylcellulose and xylan) impact these microbiomes. Furthermore, sub-enriched microbiomes were selected to evaluate their capacity to produce hydrolytic enzymes using a low-effective medium (i.e., a blend of SMS and digestate). Secretomes were analysed in terms of their optimal temperature and pH, thermal stability (including shelf-life at 4 °C), and the types of products generated upon the breakdown of pure hemicellulose-derived polymers.

Our results revealed distinct microbial proliferation patterns based on temperature regimes with a higher initial biomass at 37 °C, reflecting the dominance of mesophilic microbes in the initial inoculum. Temperature strongly influenced substrate utilization, with varied preferences for cellulose, hemicellulose, and lignin degradation across different temperature regimes. The microbiome selected at 37 °C showed significant degradation across all three components, while those at 50 °C and 70 °C exhibited preferences for hemicellulose and lignin. Thermal and elemental analyses supported these findings, indicating differential degradation of cellulose, hemicellulose, and lignin at different temperatures. Enzyme activity assays confirmed the presence of lignocellulolytic enzymes in the culture supernatants, correlating with substrate degradation trends. Moreover, thermogravimetric analysis can be a more time-saving approach (1 h per sample at 10 °C/min) for the determination of the saccharification potential (i.e., the sum of cellulose and hemicellulose that can be converted to fermentable sugars) than total carbohydrate analysis (TCA) that takes 2–3 days per sample.

The isolation and characterisation of microbiomes led to the discovery of thermophilic and thermostable xylanases. These enzymes, produced by microbiomes enriched at 37 °C (namely, CMC-37 and XYL-37), exhibited distinct temperature and pH optima, along with considerable thermostability, especially when incubated below their temperature optima. Notably, these xylanases demonstrated more robust stability than a commercial enzyme mixture (i.e., Cellic^®^ CTec2), especially under conditions resembling their natural environment in the digestate. The study highlighted the potential of these enzymes for biomass saccharification, as their temperature optima aligned with those used in lignocellulose biorefineries. Electrophoretic analyses revealed complex protein profiles, indicating the presence of numerous xylanases. Zymography further identified multiple xylanase bands in both microbiomes, with XYL-37 showing a greater diversity of xylanases. Quantification of enzyme units demonstrated substantial xylanase activity in both secretomes, suggesting their potential for enzymatic hydrolysis despite being cultured in a medium solely composed of industrial leftovers. Substrate profiling confirmed catalytic activity on various polysaccharides, showcasing their versatility in hydrolysing different hemicellulose-derived polysaccharides.

The exploration of microbial consortia composition through PCR-DGGE and metagenomic analyses unveiled intricate shifts in microbiome dynamics under different environmental conditions. The rapid divergence in microbiome composition following substrate and temperature variations was striking and was especially evident in the drastic dissimilarity observed in cultures incubated at 37 °C, 50 °C, and 70 °C compared to the initial inoculum. This suggests the swift adaptation of microbial populations to the provided conditions, particularly stabilizing at 37 °C and 50 °C while facing challenges at 70 °C due to the limited selection of lignocellulolytic microbes. The dominance of thermophilic bacteria at higher temperatures signifies a preferential proliferation of organisms adapted to extreme conditions, a pattern reflected in DGGE profiles. Metagenomic data enriched our understanding, providing a more comprehensive view of microbial composition and functional potential. The taxonomic classification using kraken2 highlighted variability among the microbiomes, showcasing dominant phyla shifts corresponding to specific substrates and temperatures. Furthermore, metagenome assembly and binning revealed varying complexity across datasets, leading to the identification of metagenome-assembled genomes (MAGs). Notably, the number of MAGs retrieved from different microbiomes emphasised the influence of environmental factors on microbial community richness. The meta-functional analysis of carbohydrate-active enzymes (CAZymes) sheds light on the microbiomes’ metabolic potential. The prevalence of GHs indicated their pivotal role in carbohydrate substrate hydrolysis, with GH13, GH4, and GH31, among others, linked to starch degradation. The identification of cellulose- and hemicellulose-degrading enzyme families highlighted microbial strategies for lignocellulose biomass transformation. Variations in the abundance of ORFs encoding these enzymes across different microbiomes underscored the impact of selective growth conditions, with temperature and substrate specificity dictating the degradative potential of the communities. Observations on pectin-degrading enzymes and lignin deconstruction further emphasised the multifaceted nature of microbial enzymatic activity in lignocellulose degradation. Notably, the reduction in lignin-degrading CAZymes under certain conditions hinted at factors constraining the microbiome’s degradative potential, indicating a complex interplay between environmental factors and microbial community structure.

This study provides valuable insights into temperature-driven microbial selection for lignocellulose degradation, resulting in the discovery of thermophilic xylanases with favourable characteristics for industrial applications. The findings hold promise for optimizing enzyme production and formulating enzyme mixtures for efficient biomass conversion in biorefineries. Overall, this study manuscript offers a comprehensive understanding of the dynamics, characteristics, and potential applications of lignocellulolytic microbial consortia, presenting promising avenues for sustainable bioconversion processes. Moreover, our findings provide a comprehensive understanding of how microbial consortia respond to variations in temperature and substrate constraints. The intricate shifts in microbial composition and functional potential elucidate the complex dynamics governing lignocellulose degradation and highlight avenues for further exploration in optimizing bioconversion processes. The observed alterations in microbial communities and their enzymatic repertoire emphasise the need for tailored strategies to harness their potential in biotechnological applications.

## 4. Materials and Methods

### 4.1. Spent Mushroom Substrate and Microbial Inoculum Characterisation

Spent mushroom substrate (SMS) was collected from the agro-zootechnical farm “La Torre”, located in Isola della Scala (Verona, Italy). SMS was oven-dried at 105 °C for about 16 h, ground, and passed through a 2 mm sieve.

Total carbon (C), nitrogen (N), hydrogen (H), and sulphur (S) concentrations in bulk and incubated SMS samples, as well as in the digestate sample, were determined by dry combustion using a CHNS macro analyser (vario Macro cube, Elementar, Langenselbold, Germany). Samples were analysed in triplicate. Alfalfa organic analytical standard (OAS) (B2273, Elemental Microanalysis Limited, Okehampton, UK) was used as reference material.

SMS samples, before and at the end of the enrichment cultures, were also analysed using a thermogravimetric (TG) analyser coupled with simultaneous differential scanning calorimetry (DSC) (TGA-DSC 3+, Mettler Toledo, Switzerland). An aliquot of ca. 30 mg of each sample was placed in an alumina crucible and heated from 30 to 700 °C at 10 °C/min under air at a flow rate of 100 mL/min. The weight losses (WL) occurring over the exothermic region 105 to 550 °C were also normalised by the loss-of-ignition (LOI). Weight losses occurring over the temperature range 250–350 °C, associated with sugars and cellulosic materials, and 350–450 °C, associated with the degradation of more recalcitrant structures including lignin [36,41], were also determined.

For the enrichment cultures, the digestate was obtained from the anaerobic digestor of the above-mentioned farm, which operated under mesophilic conditions. The digestate was passed through a 2 mm sieve (Giuliani Tecnologie, Torino, Italy) and characterised for total solids (TS) and total volatile solids (TVS) [42,43] (Table 5).

### 4.2. Media and Chemicals

Agarised Luria–Bertani medium (LB-agar, also known as lysogeny broth) was used for the colony-forming unit count (CFU) of cultivations at 37 and 50 °C and contained 1% (*w*/*v*) NaCl (Fluka Honeywell, Charlotte, NC, USA), 1% (*w*/*v*) tryptone (VWR Chemicals, Milano, Italy), 0.5% (*w*/*v*) yeast extract (VWR Chemicals, Milano, Italy), and 1.5% (*w*/*v*) agar. For cultivations at 70 °C, LB-agar (same as above) was supplemented with the following additional chemicals (*w*/*v*): 0.2% (NH_4_)_2_SO_4_, 0.4% Na_2_HPO_4_, 0.25% NaH_2_PO_4_, 0.024% MgCl_2_, 0.008% CaCl_2_, 0.0062% NH_4_Cl, 0.001% FeSO_4_, 0.0026% KCl, trace elements, and vitamins [44].

The solid medium used for the further enrichment (i.e., sub-enrichment) of the microbial consortia contained a final concentration of 1.5% (*w*/*v*) agar (Neofroxx, Pozzuoli, Italy) and either 1% carboxymethylcellulose (CMC, VWR Chemicals, Milano, Italy) or 1% corncob xylan (TCI Chemicals, Shanghai, China). The medium was added with the above-mentioned additional components for cultivations at 70 °C.

For enzyme secretion, a liquid medium composed of 0.5% (*w*/*v*) yeast extract and either 0.5% CMC or corncob xylan was used to revitalise glycerol stocks for enzyme production (seed medium). The induction medium was composed of 5% (*w*/*v*) sterile MSM in sterile digestate, previously centrifuged at 6000× *g* for ten minutes to remove solid particles, and tenfold diluted. 

### 4.3. Enrichment Cultures

To select lignocellulose-degrading microorganisms, the coarsely filtered digestate was used as microbial inoculum for enrichment batch cultures on SMS as a lignocellulose substrate. Precisely 16.8 g of SMS was mixed with 36.5 mL of digestate, and the volume was adjusted to 300 mL with dH_2_O. Three enrichment cultures were set up and incubated, respectively, at 37, 50, and 70 °C in an Innova^®^ 42, Eppendorf (New Brunswick, Edison, NJ, USA) in 1 L baffled Erlenmeyer flasks. The cultures were kept under a constant shaking rate of 180 rpm.

Enrichment cultures were assessed daily by monitoring (i) microbial proliferation and (ii) endo-1,4-β-D-xylanase and endo-1,4-β-D-glucanase activities in the culture supernatant, while (i) residual content of cellulose, hemicellulose, and lignin, and (ii) elemental (CHNS) and thermal (TG-DSC) analyses were carried out at T_0_ as well as at the end of the enrichment cultures (T_f_). Total carbohydrate analysis (TCA) was performed at the beginning (T_0_) and at the end of the enrichment (T_9_). Total carbon (C), nitrogen (N), hydrogen (H), and sulphur (S) concentrations in bulk and incubated SMS samples, as well as in the digestate sample, were determined by dry combustion using a CHNS macro analyser. Samples were analysed in triplicate. Alfalfa OAS (B2273, Elemental Microanalysis Limited) was used as the reference material. Genomic DNA extraction was carried out at T_0_ as well as after three (T_3_), six (T_6_), and nine (T_9_) days.

**Figure 14:**
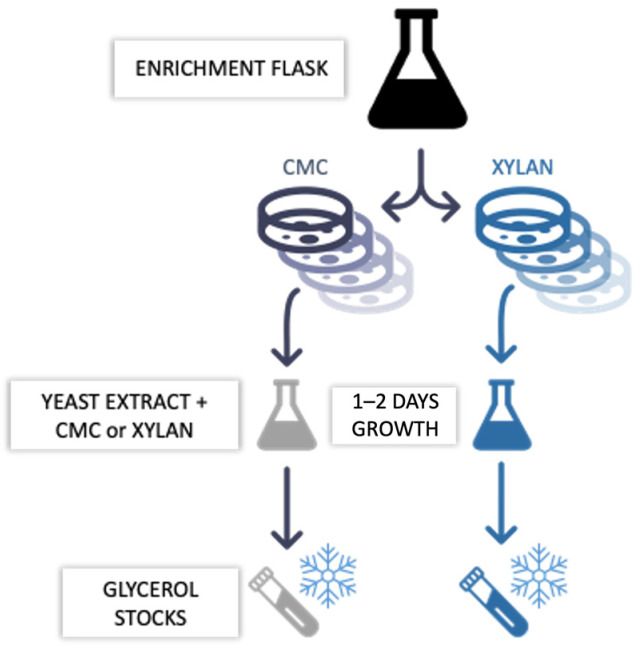
Scheme of the microbiomes’ sub-enrichment on pure polymeric carbon sources (i.e., CMC or xylan). Samples were withdrawn from the main enrichment flasks and seeded on solid media containing either 1% CMC or xylan to further select status (i.e., sub-enriched) and incubated at the respective enrichment temperatures. Upon appearance of a mat of colonies on the plate’s surface, sub-enriched consortia were recovered, proliferated in liquid medium, and then stored as glycerol stocks at −80 °C.

Throughout the enrichment, every other day, each culture was seeded on solid media containing either 1% CMC or xylan to further select (i.e., sub-enriched) for cellulolytic (i.e., CMC-37, CMC-50, and CMC-70) or hemicellulolytic (i.e., XYL-37, XYL-50, and XYL-70) subpopulations (i.e., microbial consortia). The plates were incubated at 37 °C, 50 °C, or 70 °C, matching the temperatures of the enrichment, until a mat of colonies appeared on their surface, generally between 1 and 7 days. To avoid drying the plates, they were sealed in plastic bags together with wetted paper strips. First, the sub-enriched consortia were resuspended in 1% (*w*/*v*) NaCl solution. Then, the cells were pooled together based on their temperature and substrate of sub-enrichment. Next, each pool was inoculated in 50 mL of liquid medium containing 0.25% (*w*/*w*) yeast extract and 0.5% (*w*/*v*) CMC for CMC-37, CMC-50, and CMC-70 or 0.5% (*w*/*v*) xylan for XYL-37 and XYL-50. It was not possible to isolate a consortium of XYL-70 because no proliferation was detected on the sole xylan at 70 °C. The five microbial consortia were kept under a constant shaking rate of 180 rpm at their respective temperature of enrichment. Lastly, each consortium was pelleted at 3000× *g* for five minutes (Eppendorf 5430R5430R), and the pellets were resuspended in a 25% (*v*/*v*) glycerol (Biochem Chemopharma, Cosne-Cours-sur-Loire, France) solution and stored at −80 °C (Figure 14).

#### 4.3.1. Counting of Colony-Forming Units

Microbial growth was assessed via CFU count on LB-agar plates. Withdrawn samples were serially diluted (tenfold). In brief, 1 g of each enrichment culture was supplemented with a solution of 1% (*w*/*v*) NaCl up to 10 mL before serial dilutions. Then, 100 µL of each dilution was plated on the surface of LB-agar plates and incubated, between 1 and 7 days, at the same temperature as the enrichment.

#### 4.3.2. Endo-1,4-β-D-Xylanase and Endo-1,4-β-D-Glucanase Assays

The presence of endo-1,4-β-D-xylanase and endo-1,4-β-D-glucanase in the culture supernatants was tested every day by means of enzyme assays, using Azo-Xylan and Azo-CMC as substrates, respectively. Enzyme assays were carried out following the manufacturer’s instructions (Megazyme, Bray, Ireland), except for a few modifications. Briefly, 50 μL of aliquots of 1% *w*/*v* Azo-Xylan or Azo-CMC solutions and 50 μL of aliquots of cell-free culture supernatants were preincubated for 5 min at the desired assay temperature (i.e., 37 °C, 50 °C, or 70 °C). To start the reactions, prewarmed Azo compounds (i.e., the substrates) and supernatants (i.e., the enzymes) were mixed and incubated for two hours at the desired temperature. Then, the reactions were stopped by adding 250 μL of precipitating solution and left for 10 min at room temperature. In parallel, negative controls were prepared by directly mixing the precipitating solution and the Azo-Xylan or Azo-CMC solutions before adding the culture supernatant. Next, all the samples were centrifuged for 5 min at 10,000× *g* to remove the nonhydrolysed polymeric substrate, and 200 μL of clarified reaction mixture was transferred into a well of a 96-well microplate (Sarstedt, Nümbrecht, Germany). The xylanase or cellulase activity was determined, following the assay manufacturer guidelines, with reference to a standard curve (Cellic Ctec2 Novozymes, Bagsværd, Danimarca) by measuring the absorbance at 590 nm using a Synergy Neo2 Hybrid Multi-Mode Reader (Agilent Technologies, Santa Clara, CA, USA). All assays were run in triplicate.

#### 4.3.3. Total Carbohydrates and Lignin Analysis 

Variations in cellulose, hemicellulose, and lignin content of the SMS were determined using the National Renewable Energy Laboratory “Determination of Structural Carbohydrates and Lignin Biomass” protocol with minor modifications [45]. Briefly, 10 g of the enrichment cultures (T_0_ and T_f_) were collected and centrifuged at 5000× *g* for 5 min. Then, the pellet was washed with a 1% NaCl solution, alternating cycles of pelleting the insoluble solids and resuspension with fresh 1% NaCl until clear. After exsiccation of the samples at 45 °C for 24 h, the water-insoluble solid (WIS) was ball milled at 30 Hz for 1 min by means of a Mixer mill MM 400 (Retsch, Haan, Germany). Then, 60 mg of WIS was accurately weighed and resuspended in 600 µL of 72% (*w*/*w*) H_2_SO_4_ inside a glass tube provided with a Teflon cap. The mixture was incubated for 60 min at 30 °C, frequently stirring to result in homogenised hydrolysis. Next, the samples were diluted up to 4% H_2_SO_4_ using OdH_2_O and were autoclaved for 60 min at 121 °C. Once the hydrolysates cooled down, the samples were filtered on a preweighed nylon membrane with 0.22 µm cut-off in a filtering bottle apparatus (LLG, Meckenheim Germany). The membranes were washed extensively with dH_2_O and were dried at 70 °C for 48 h to determine acid-insoluble residue (AIR) and acid-insoluble lignin (AIL) contents (Equations (1) and (2)). An aliquot of the filtrate was used for acid-soluble lignin (ASL) determination at 205 nm with the use of a spectrophotometer (LLG, Meckenheim, Germany) (Equation (3)). The remaining permeate was neutralised with the addition of CaCO_3_. Then, the samples were centrifuged at 5000× *g* for 5 min. The supernatants were filtered through a 0.22 μm polytetrafluorethylene membrane and stored at −20 °C following HPLC determination of glucose, xylose, and arabinose concentrations. A set of sugar recovery standards (SRS) for glucose, xylose, and arabinose were used as controls for the loss of sugars during the dilute acid hydrolysis step and included in the calculation of cellulose and hemicellulose content (Equations (5) and (6)). The content of lignin was calculated using Equation (4). All samples were prepared in triplicate, and the results were shown as the mean ± SD.

(1) $$\%\;AIR=\frac{Weight_{membrane+sample}-Weight_{membrane}\;}{Weight_{sample}\times \;\%\;TS/100}\times 100$$

(2) % AIL=% AIR−% ashes

(3) $$\%\;ASL=\frac{UV_{abs}\times V_{filtrate}\times Dilution\;factor}{110\times \left(Weight_{sample}\times \%\;TS/100\right)}\times 100$$

(4) % Lignin=%AIL+% ASL

(5) $$\%\;Cellulose=\frac{C_{glu}\times 100\times 0.9\times Volume_{filtrate}\;}{Recovery_{SRS}\;\%\times \left(Weight_{sample}\times \%\;TS/100\right)}\times 100$$

(6) $$\%\;Hemicellulose=\frac{\left(C_{xil}+C_{ara}\right)\times 100\times 0.88\times Volume_{filtrate}\;}{Recovery_{SRS}\;\%\times \left(Weight_{sample}\times \%\;TS/100\right)}\times 100$$

### 4.4. Microbial Consortia Cultivations for Enzymes Production

Microbiomes selected at 37 °C on CMC and xylan were revitalised in 50 mL of seed medium and incubated at the temperature of enrichment under a constant shaking rate of 180 rpm. After overnight incubation, each sample was reinoculated in triplicate in the induction medium at 0.05 OD_600_/mL and incubated in the same conditions. Two noninoculated controls were prepared to assess the sterility of both the SMS and the digestate. Microbial growth was assayed by CFU counting (see Section 4.3.1). Samples were collected at time zero (T_0_) and every 24 h. Cell-free supernatants (secretomes), obtained by centrifugation at 6000× *g* for 5 min, were subjected to colourimetric enzyme assay using Azo-Xylan as the substrate (see Section 4.3.2). After the enzymatic activity reached a plateau, 10 mL of the culture that had reached the highest xylanase activity within the triplicates was used to seed three shake flasks each containing 100 mL of induction medium. A maximum in the xylanase activity was generally detected again after two days of cultivation. Then, the cultures were centrifuged twice at 6000× *g* for 10 min to remove the bulk of the remaining SMS and filtered through a 0.22 μm nylon membrane by means of a vacuum-filtration apparatus (LLG-Labware, Meckenheim, Germany). Enzymatic activity of the secretomes was characterised in terms of temperature and pH optima, thermal stability, and types of products released from beechwood xylan (P-XYLNBE, Megazymes, Bray, Ireland), wheat flour arabinoxylan (P-WAXYM, Megazymes, Bray, Ireland), tamarind xyloglucan (P-XYGLN, Megazymes, Bray, Ireland), and ivory nut mannan (P-MANIV, Megazymes, Bray, Ireland) (see Section 4.6).

### 4.5. Reducing Sugar Assay to Measure the Enzyme Units 

The 3,5-dinitrosalicylic acid (DNS) assay was used to measure the concentration of reducing sugars following the production of 3-amino,5-nitrosalicylic acid (ANS) at 570 nm [46,47]. The DNS solution was prepared by adding 4 g of NaOH (Fluka Honeywell, Charlotte, NC, USA) to 50 mL of dH_2_O. Then, 0.25 g of DNS (Thermo Fisher, Rodano, Italy) and 75 g of potassium sodium tartrate (VWR Chemicals, Milano, Italy) were added to the alkaline solution. Finally, the volume was brought to 250 mL with dH_2_O. The solution was sterilised through filtration using a 0.22 μm PES filter. The DNS assay was carried out by mixing the same volumes of secretome and 1% (*w*/*v*) xylan (P-XYLNBE, Megazyme, Bray, Ireland) solution for a total of 70 μL. To account for the presence of sugars in the culture supernatant and/or in the xylan solution, two control samples were prepared. An enzyme control was prepared by mixing the secretome and dH_2_O, whereas the substrate control was prepared by mixing the same volumes of the xylan solution and dH_2_O. Samples were incubated at the desired temperature for 10 min under a constant shaking rate (800 rpm). To stop the reactions, 164 μL of DNS reagent was added to each tube and samples were incubated at 100 °C for 20 min in a thermomixer. Then, samples were cooled down for 10 min on ice and briefly centrifuged. Finally, absorbance was measured at 570 nm by means of a Synergy Neo2 multimode microplate reader (BioTek, Milano, Italy). A calibration curve was prepared using different xylose concentrations ranging from 0.5 mg/mL to 4 mg/mL. The enzyme activity (U/mL) was calculated by means of the following equation:

$$\frac{U}{mL}=\frac{\left[released\;xylose\;(\mu mol)\right]}{\left[reaction\;time\left(min\right)\right]\times \left[enzyme\;volume\left(mL\right)\right]}$$

### 4.6. Hydrolysis of LCB-Derived Polysaccharides

Beechwood xylan (P-XYLNBE, Megazyme, Bray, Ireland), wheat flour arabinoxylan (P-WAXYL, Megazyme, Bray, Ireland), tamarind xyloglucan (P-XYGLN, Megazyme, Bray, Ireland), and ivory nut mannan (P-MANIV, Megazyme, Bray, Ireland) were used as substrates to test secretome enzymatic activities. Substrates were mixed with the secretomes at the final concentration of 1% (*w*/*v*). Samples were incubated under a constant shaking rate (800 rpm) using an LLG-uniTHERMIX2 pro thermo shaker (LLG. Meckenheim Germany). The digestion temperature and pH were set at the optimal value for each secretome. Hydrolysis was stopped after 1, 2, 3, 4, 6, and 24 h by boiling the reaction at 100 °C for 5 min to inactivate the enzymes.

### 4.7. High-Performance Anion-Exchange Chromatography (HPAEC) with Pulsed Amperometric Detection (PAD)

HPAEC-PAD was carried out on a Dionex ICS-6000 system equipped with a CarboPac PA210-Fast-4 μm column, a palladium hydrogen (PdH) reference electrode, and a gold working electrode for detection. The flow rate was set to 0.6 mL/min. Elution of mono- and oligosaccharide was achieved by a gradient (from 12 mM to 100 mM) of potassium hydroxide. The auto-sampler was set at 10 °C while the column was at 30 °C. Samples were centrifuged (10,000× *g* for 5 min), appropriately diluted with dH_2_O, and filtered using 0.22 μm syringe filters. Finally, monosaccharides (Sigma-Aldrich, Saint Louis, MI, USA) and oligosaccharides (Megazyme, Bray, Ireland) were identified and quantified through standard curves prepared in the range 25–0.1 mg/L. All experiments were repeated in triplicate and the error was calculated as the standard deviation of the three measurements.

### 4.8. Electrophoretic Analysis and Zymography

Semi-denaturing sodium dodecyl sulfate–polyacrylamide gel electrophoresis (SDS-PAGE) was performed following Laemmli’s method [48], with minor modifications. Samples were mixed with a nonreducing loading buffer (1.5 M Tris-HCl pH 6.8, 8% SDS, 40% glycerol, bromophenol blue) and were not subjected to boiling denaturation. The protein marker spanned from 10 to 250 kDa (Precision Plus Protein Standard Unstained, Biorad, Segrate, Milan, Italy). For zymography, beechwood xylan 1% (*w*/*v*) (P-XYLNBE, Megazyme, Bray, Ireland) was added to the resolving gel during the preparation of the acrylamide matrix. Following electrophoresis, the gel was washed with a 2.5% (*v*/*v*) Triton X-100 solution for 30 min at 4 °C, followed by a 30 min incubation at 4 °C in the desired assay buffer. The reaction is performed through an incubation at the enzyme’s optimum temperature. The gel is then stained with 0.1% (*w*/*v*) Congo Red solution for 30 min at room temperature. Areas of digestion appeared as clear bands against a darkly stained background after destaining by washing with 1M NaCl for 15 min.

### 4.9. Total DNA Extraction, PCR Reaction, and DGGE (Denaturing Gradient Gel Electrophoresis) Analysis

Microbial communities’ composition was evaluated by polymerase chain reaction–denaturing gradient gel electrophoresis (PCR-DGGE). Total DNA was obtained through FAST DNA^®^ Spin Kit for Soil (MO BIO, Carlsbad, CA, USA), following the manufacturer’s instructions. Bacterial 16S rRNA genes were amplified by using primers fD1 and rP2 [49] (Weisburg et al., 1991), and then the amplification of the hypervariable V3 region was carried out through a nested PCR using primers p2 and p3 with a GC clamp [50] (Muyzer et al., 1993). 

Amplicons obtained from the 16S rRNA gene (~2000 ng) were loaded on 8% acrylamide/bisacrylamide gel with 30–60% denaturing gradient (100% denaturant defined as 7 M urea and 40% (*v*/*v*) formamide). The gel was electrophoresed as reported in Andreolli et al. (2020) [51]. DGGE patterns were recorded with the DCodeTM Universal Mutation Detection System (Bio-Rad; Hercules, CA, USA). Similarity indexes were calculated based on a UPGMA cluster analysis. Dendrograms were produced using the software package UVIbandmap (Uvitec).

### 4.10. Metagenomic Analyses

The analyses of the metagenomics data were performed using MetaWRAP (v1.3.2) [52], a flexible and modular pipeline that facilitates the core tasks in metagenomics analysis. The implemented workflow mainly follows the guidelines reported in the GitHub MetaWRAP repository. Sequencing reads [53] were preprocessed using the MetaWRAP-read_qc modules (default options) to trim low-quality bases, clip the adapter sequences, and remove possible human contaminant reads. Preprocess reads were at first quickly classified using kraken2 (v2.0.9-beta) [54], as implemented in the MetaWRAP_kraken2 module, against a database containing archaea, bacteria, fungi, viral, and human genomes retrieved from the GenBank database.

Visual representations of the reads’ classification were performed with the Krona tool (v2.7) [55]. Metagenome assembly was performed for each sample independently using MegaHit (v1.1.3) [56] implemented in the MetaWRAP-Assembly module. The assemblies produced for each sample were then binned using the MetaWRAP-Binning module using three different binning software, MaxBin2 (v2.2.6) [57], metaBAT2 (v2.12.1) [58], and CONCOCT (v1.0.0) [59]. The obtained bins were further validated and refined using the MetaWRAP-BIN_refinement module, which consolidates multiple binning predictions into a new and improved bin set. The module was run setting a minimum percentage of genome completeness equal to or higher than 50% and with no more than 5% of contamination, as calculated by CheckM (v1.0.12) [60]. To obtain a final list of nonredundant bins across all the samples, bin sequences were dereplicated using the dRep program (v3.4.0) [61]. Bin quantification was performed using the MetaWRAP-Quant_bins module, providing, as a reference (option-a), the fasta file with the entire metagenomic assembly. Bin taxonomy assignment was performed using GTDB-Tk (v2.1.1) [53], a software toolkit developed to assign objective taxonomic classifications to bacterial and archaeal genomes based on the Genome Database Taxonomy [62].

Gene prediction was performed using prodigal software (v2.6.3) [63], while gene functional annotation was completed by means of eggnog-mapper v2 [64]. The 131 dereplicated microbial genomes were imported in the KBase system (on 10 January 2023) to create a genome set using Batch Create Genome Set v1.2.0 and annotated with RASTtk v1.073. The CAZymes were annotated with the tool Search with dbCAN2 HMMs of CAZy families v10. 

## Figures and Tables

**Table 1:** Enzyme units in the secretomes CMC-37 and XYL-37.

Secretome Name	Units/mL
CMC-37	2.29 ± 0.20
XYL-37	0.47 ± 0.02

**Table 2:** Metagenome assemblies’ metrics.

	Contigs	Contigs (≥5 Kb)	N50	L50
T_0_-SMS	52,084	5182	3538	8157
T_9_-37	65,657	7924	6133	6137
T_9_-50	39,024	5777	10,431	2652
T_9_-70	13,240	1959	8148	1135
CMC-37	6257	1174	12,179	422
CMC-50	247	50	365,423	5
CMC-70	37	29	360,411	4
XYL-37	2790	697	10,645	230
XYL-5050	2274	630,630	2,374,023,740	155,155

**Table 3:** Binning results.

	# MAGs	# Contigs in MAGs	# Total Contigs	% Contigs in MAGs
T0-SMS	38	12,744	52,084	24.5
T_9_-37	39	14,065	65,657	21.4
T_9_-50	32	14,983	39,024	38.4
T_9_-70	10	3115	13,240	23.5
CMC-37	7	4024	6257	64.3
CMC-50	1	100	247	40.5
CMC-70	1	21	37	56.8
XYL-37	1	109	2790	3.9
YXYL-50	3	788	2274	34.7

**Table 4:** CAZyme distribution in all metagenomes. The total number of genes (for each class) has been derived from the dbCAN2 tool.

Metagenomes	CAZyme Classes					
	GH	GT	CE	AA	PL	CBM
**T_0_-SMS**	1335	612	246	29	52	53
**T_9_-37**	1477	1109	352	151	53	76
**T_9_-50**	1099	879	205	132	55	53
**T_9_-70**	390	288	92	21	5	25
**CMC-37**	280	152	32	22	8	3
**CMC-50**	59	28	11	4	8	4
**CMC-70**	32	16	6	4	0	2
**XYL-37**	157	77	15	10	12	2
**XYL-50**	85	57	29	10	7	4
**No. Sequences**	4914	3218	988	383	200	222
**Relative abundance (%)**	49.5	32.4	10.0	3.9	2.0	2.2

**Table 5:** SMS and inoculum characterization.

	^a^ SMS	Digestate
TS (% *w*/*w* f.m.)	94.99 ± 2.18	7.04 ± 0.01
TVS (% *w*/*w* f.m.)	81.17 ± 1.97	4.11 ± 0.03
C (% *w*/*w* d.w.)	38.64 ± 0.11	29.55 ± 0.19
N (% *w*/*w* d.w.)	0.71 ± 0.01	3.00 ± 0.08
S (% *w*/*w* d.w.)	0.51 ± 0.01	1.13 ± 0.01
H (% *w*/*w* d.w.)	5.72 ± 0.05	4.11 ± 0.05
C/N	54.43 ± 1.11	9.85 ± 0.31
Cellulose (% *w*/*w*)	34.24 ± 0.71	–
Hemicellulose (% *w*/*w*)	19.45 ± 0.31	–
Lignin (% *w*/*w*)	17.73 ± 0.98	–
Ash (% *w*/*w* f.w.)	13.81 ± 0.21	2.93 ± 0.02

**^a^** Spent mushroom substrate; fresh weight (f.w.); dry weight (d.w.); not measured (–).

## Data Availability

Sequencing data have been submitted to SRA database, project PRJNA1047304.

## Supplementary Materials

The following supporting information can be downloaded at: https://www.mdpi.com/1422-0067/25/2/1090/s1.
