# Supplementary material for: Lignocellulolytic Potential of Microbial Consortia Isolated from a Local Biogas Plant: The Case of Thermostable Xylanases Secreted by Mesophilic Bacteria
Source: Int J Mol Sci. 2024 Jan 16;25(2):1090. doi: 10.3390/ijms25021090 (PMC10816813; doi:10.3390/ijms25021090)
Supplement: Supplementary file 1 [file ijms-25-01090-s001.zip › Supplementary Files/Table S2.docx]

**Table S2.** HPAEC-PAD quantification of arabinoxylan hydrolysis products obtained using the secretome of the consortium XYL-37.

| **Sample Name** | **Arabinose**  g/L ± S.D. | **Xylose**  g/L ± S.D. | **Xilobiose**  g/L ± S.D. | **Xilotriose**  g/L ± S.D. | **Xilotetraose**  g/L ± S.D. |
| --- | --- | --- | --- | --- | --- |
| **CMC-37 Xylan (T0)** | n.d. | n.d. | n.d. | n.d. | n.d. |
| **CMC-37 Xylan (T1)** | n.d. | 0.11 ± 0.01 | 0.18 ± 0.01 | 0.33 ± 0.01 | 0.39 ± 0.01 |
| **CMC-37 Xylan (T2)** | n.d. | 0.33 ± 0.01 | 0.47 ± 0.01 | 0.70 ± 0.07 | 0.61 ± 0.07 |
| **CMC-37 Xylan (T3)** | n.d. | 0.54 ± 0.01 | 0.80 ± 0.01 | 0.85 ± 0.03 | 0.58 ± 0.02 |
| **CMC-37 Xylan (T4)** | n.d. | 0.61 ± 0.01 | 1.01 ± 0.01 | 0.82 ± 0.01 | 0.69 ± 0.17 |
| **CMC-37 Xylan (T24)** | n.d. | 2.22 ± 0.02 | 1.12 ± 0.01 | n.d. | n.d. |
| **XYL-37 Xylan (T0)** | n.d. | n.d. | n.d. | n.d. | n.d. |
| **XYL-37 Xylan (T1)** | n.d. | n.d. | 0.35 ± 0.05 | 0.43 ± 0.05 | 0.47 ± 0.05 |
| **XYL-37 Xylan (T2)** | n.d. | 0.04 ± 0.02 | 0.56 ± 0.07 | 0.63 ± 0.12 | 0.51 ± 0.06 |
| **XYL-37 Xylan (T3)** | n.d. | 0.10 ± 0.03 | 0.70 ± 0.15 | 0.61 ± 0.08 | 0.34 ± 0.07 |
| **XYL-37 Xylan (T4)** | n.d. | 0.49 ± 0.06 | 1.24 ± 0.20 | 0.63 ± 0.07 | 0.15 ± 0.01 |
| **XYL-37 Xylan (T6)** | n.d. | 0.60 ± 0.04 | 1.38 ± 0.08 | 0.79 ± 0.04 | 0.26 ± 0.04 |
| **XYL-37 Xylan (T24)** | n.d. | 1.86 ± 0.26 | 0.26 ± 0.08 | n.d. | n.d. |
| **CMC-37 AX (T0)** | n.d. | n.d. | n.d. | n.d. | n.d. |
| **CMC-37 AX (T24)** | 0.13 ± 0.01 | 0.52 ± 0.07 | 0.53 ± 0.10 | 0.07 ± 0.01 | n.d. |
| **XYL-37 AX (T0)** | n.d. | n.d. | n.d. | n.d. | n.d. |
| **XYL-37 AX (T24)** | 0.25 ± 0.01 | 0.21 ± 0.02 | n.d. | n.d. | n.d. |

n.d.: not detectable (i.e., below the limit of quantification – LOQ –)

S.D.: Standard deviations (*n* = 3)
