# Supplementary material for: Lignocellulolytic Potential of Microbial Consortia Isolated from a Local Biogas Plant: The Case of Thermostable Xylanases Secreted by Mesophilic Bacteria
Source: Int J Mol Sci. 2024 Jan 16;25(2):1090. doi: 10.3390/ijms25021090 (PMC10816813; doi:10.3390/ijms25021090)
Supplement: Supplementary file 1 [file ijms-25-01090-s001.zip › Supplementary Files/Table S1.docx]

**Table S1.** Elemental composition (CHNS), weight losses (WL) and ash content in incubated SMS samples.

|  | **T_9_-37** | **T_9_-50** | **T_9_-70** |
| --- | --- | --- | --- |
|  |  |  |  |
| C (%) | 33.76 ± 0.01 | 34.76 ± 0.02 | 37.24 ± 0.04 |
| N (%) | 2.61 ± 0.01 | 1.61 ± 0.01 | 1.17 ± 0.01 |
| H (%) | 5.11 ± 0.03 | 5.28 ± 0.00 | 5.53 ± 0.05 |
| S (%) | 0.32 ± 0.01 | 0.19 ± 0.01 | 0.40 ± 0.07 |
| C/N | 12.9 ± 0.07 | 21.7 ± 0.08 | 32.0 ± 0.16 |
|  |  |  |  |
| WL _250-350_ (%) | 42.6 | 48.4 | 53.2 |
| WL _350-450_ (%) | 18.0 | 19.7 | 21.5 |
| ash (%) | 30.1 | 25.0 | 19.0 |
