# Supplementary figures and images for: Lignocellulolytic Potential of Microbial Consortia Isolated from a Local Biogas Plant: The Case of Thermostable Xylanases Secreted by Mesophilic Bacteria
Source: Int J Mol Sci. 2024 Jan 16;25(2):1090. doi: 10.3390/ijms25021090 (PMC10816813; doi:10.3390/ijms25021090)

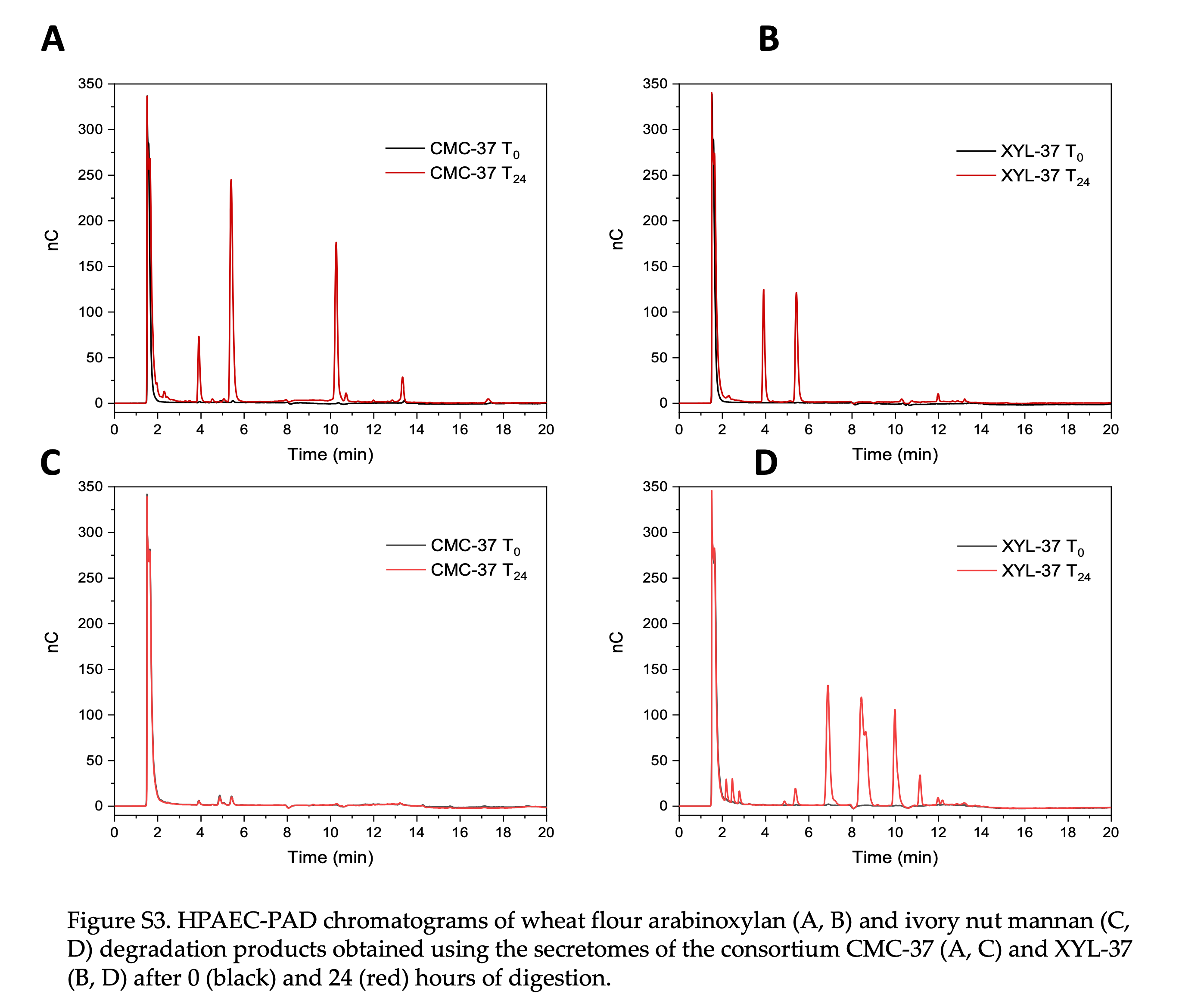

Supplement: Supplementary file 1 [file ijms-25-01090-s001.zip › Supplementary Files/Figure S3.png]

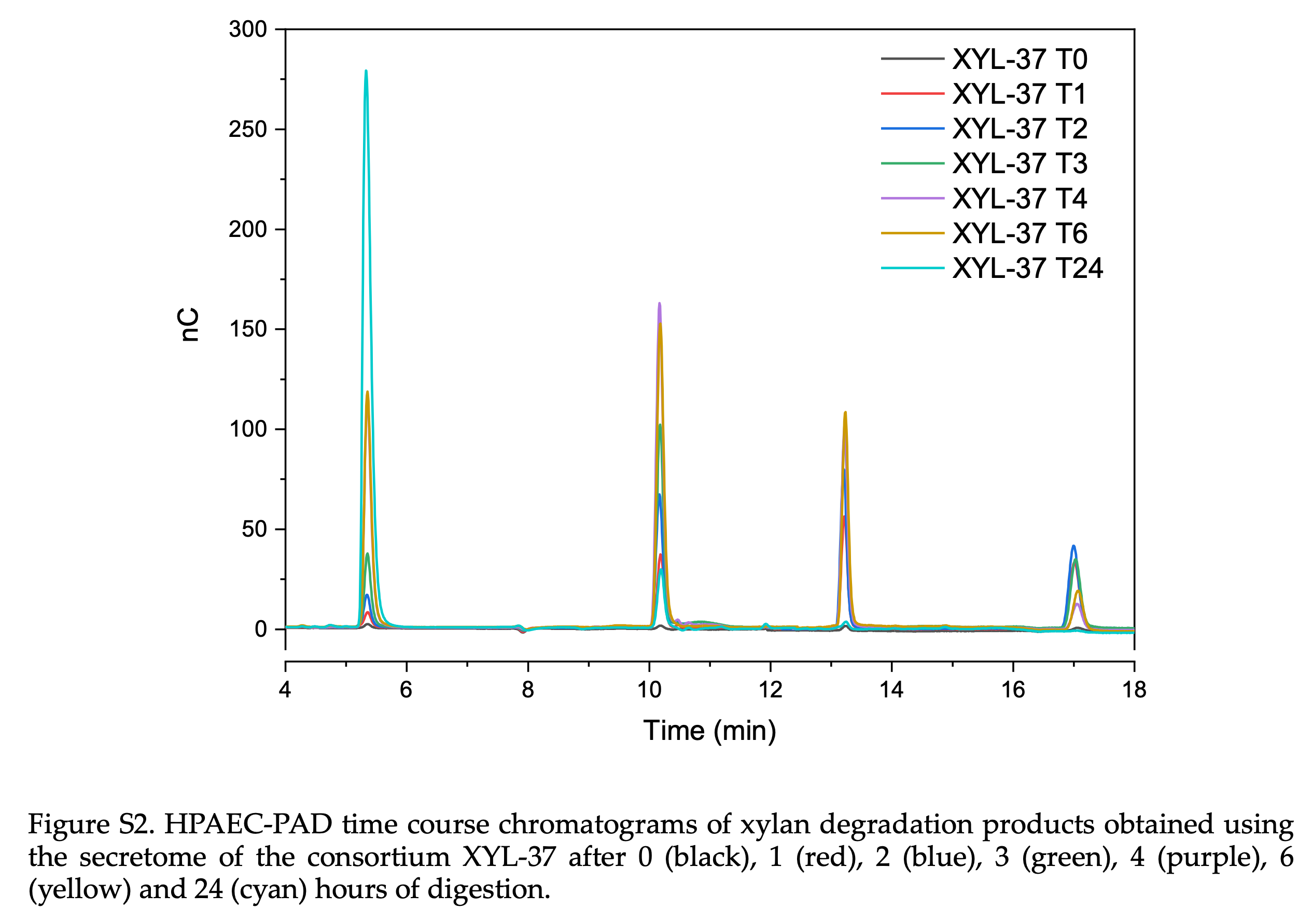

Supplement: Supplementary file 1 [file ijms-25-01090-s001.zip › Supplementary Files/Figure S2.png]

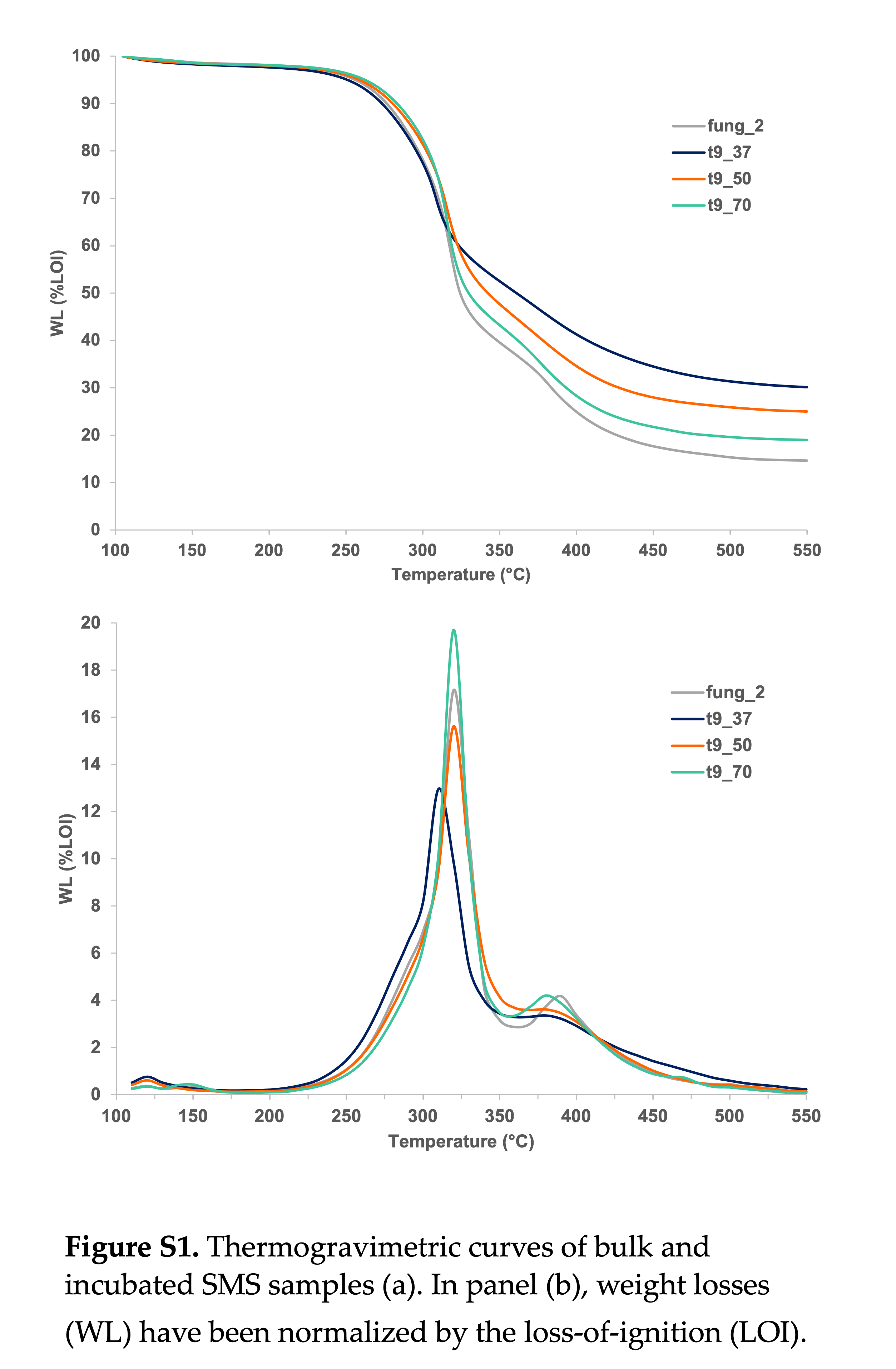

Supplement: Supplementary file 1 [file ijms-25-01090-s001.zip › Supplementary Files/Figure S1.png]

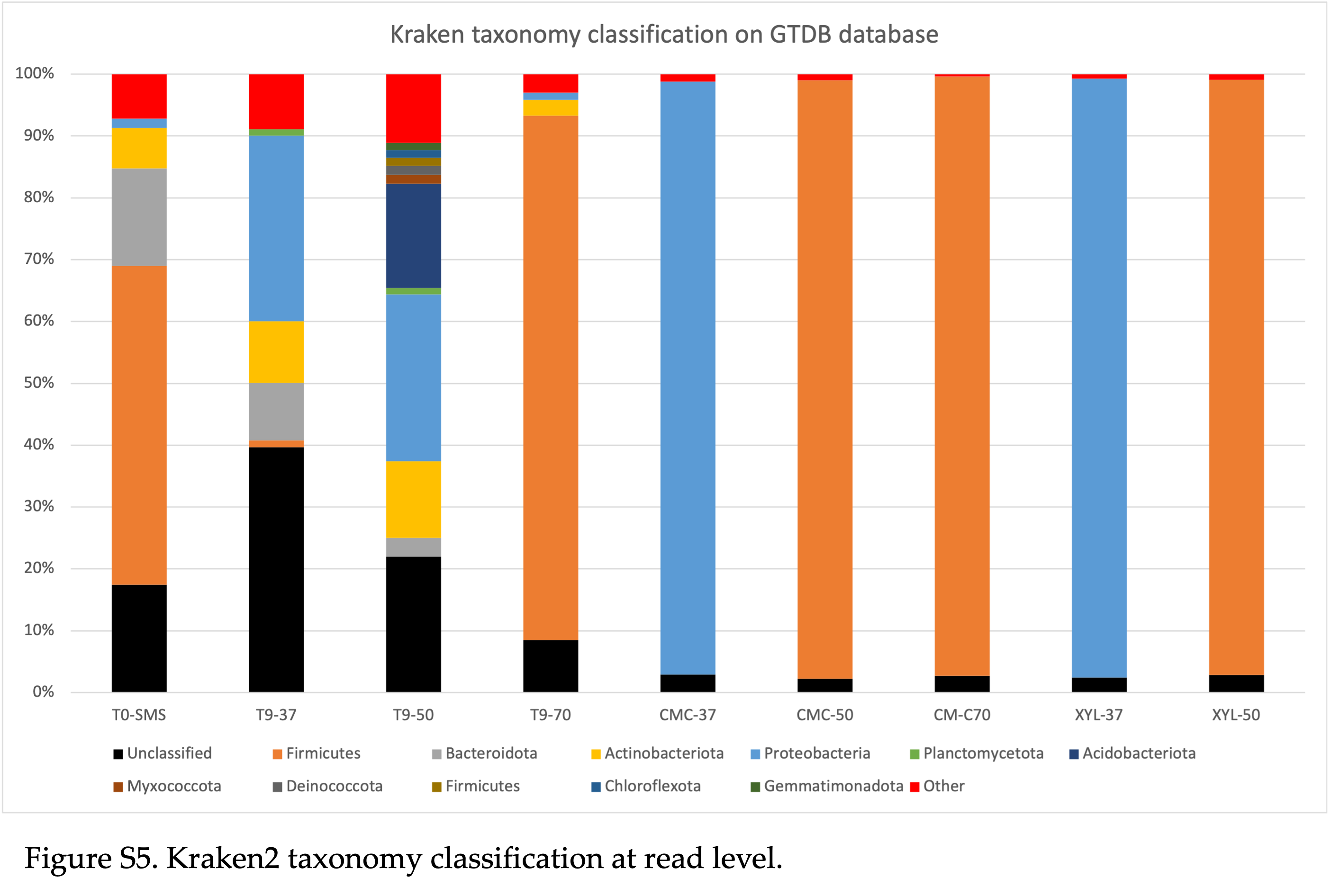

Supplement: Supplementary file 1 [file ijms-25-01090-s001.zip › Supplementary Files/Figure S5.png]

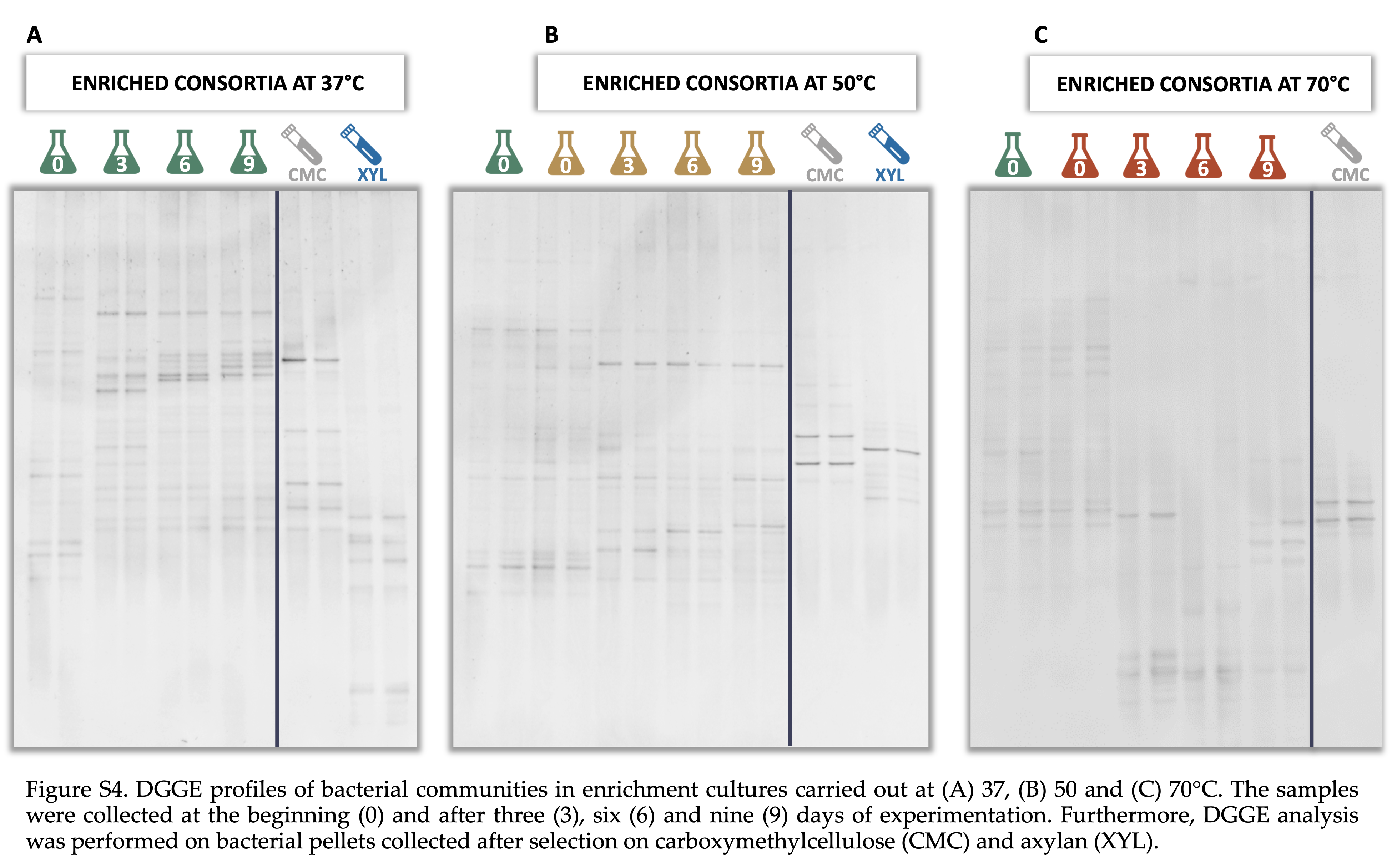

Supplement: Supplementary file 1 [file ijms-25-01090-s001.zip › Supplementary Files/Figure S4.png]

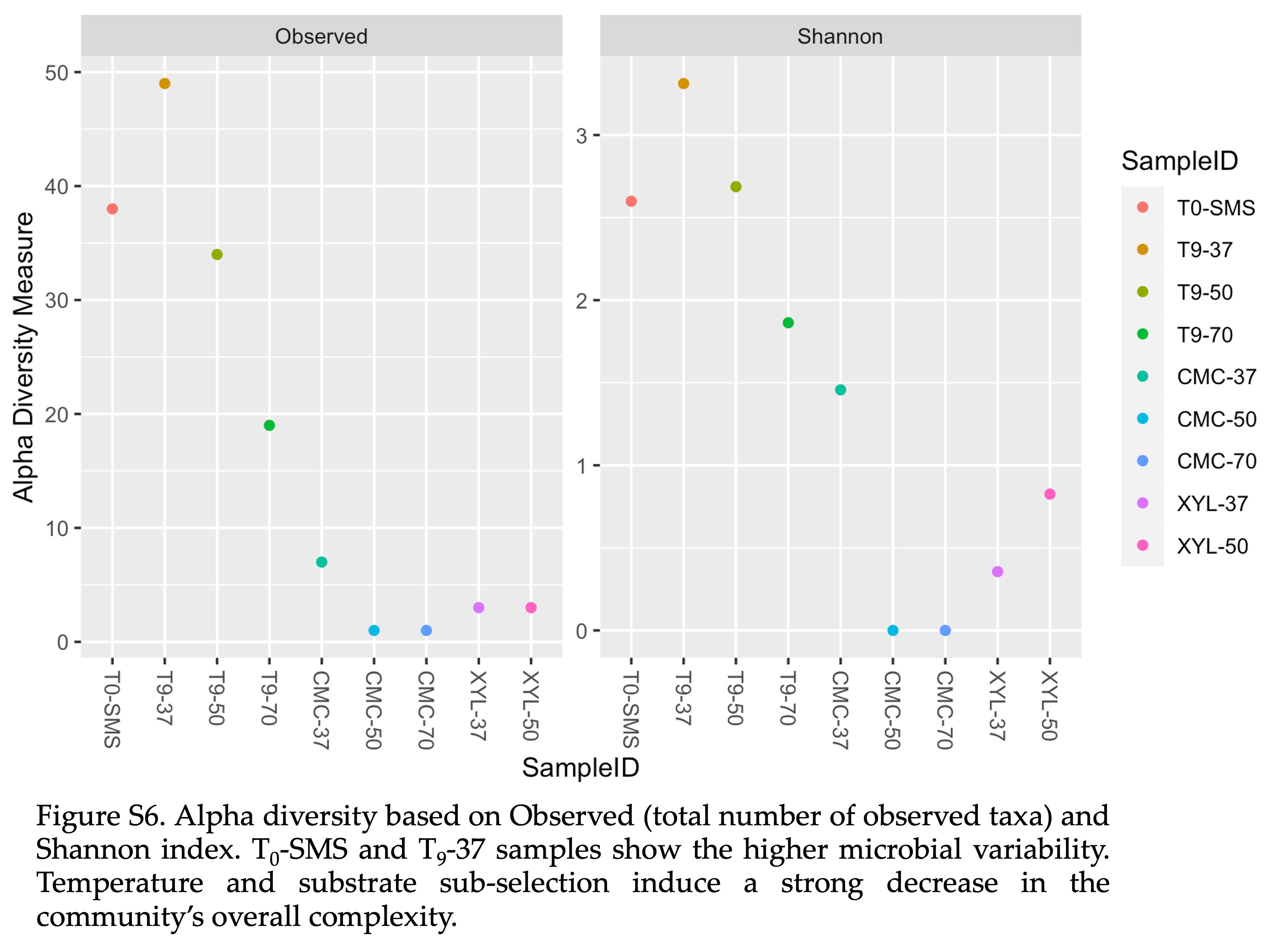

Supplement: Supplementary file 1 [file ijms-25-01090-s001.zip › Supplementary Files/Figure S6.png]
